# Biogeographical origin and timing of the founder ichthyosis *TGM1* c.1187G > A mutation in an isolated Ecuadorian population

**DOI:** 10.1038/s41598-019-43133-6

**Published:** 2019-05-09

**Authors:** U. S. Esperón-Moldes, J. Pardo-Seco, M. Montalván-Suárez, L. Fachal, M. Ginarte, L. Rodríguez-Pazos, A. Gómez-Carballa, F. Moscoso, N. Ugalde-Noritz, A. Ordóñez-Ugalde, D. Tettamanti-Miranda, J. C. Ruiz, A. Salas, A. Vega

**Affiliations:** 1 Fundación Pública Galega de Medicina Xenómica-SERGAS, Grupo de Medicina Xenómica-USC, CIBERER, IDIS, Santiago de Compostela, Spain; 2 Unidade de Xenética, Instituto de Ciencias Forenses (INCIFOR), Facultade de Medicina, Universidade de Santiago de Compostela, Galicia, Spain; 3 GenPoB Research Group, Instituto de Investigaciones Sanitarias (IDIS), Hospital Clínico Universitario de Santiago (SERGAS), Galicia, Spain; 4 Sistema de Investigación y Desarrollo SINDE, Universidad Católica de Santiago de Guayaquil and Universidad de Guayaquil, Guayaquil, Ecuador; 5 Servicio de Dermatología del Complexo Hospitalario Universitario de Santiago, Santiago de Compostela, Spain; 6 Servicio de Dermatología del Complejo Hospitalario Universitario de Vigo, Vigo, Spain; 7 Laboratorio Biomolecular, Cuenca, Ecuador; 8 Unidad de Genética y Molecular del Hospital de Especialidades José Carrasco Arteaga, Cuenca, Ecuador; 9 Universidad Espíritu Santo and Hospital Luis Vernaza, Guayaquil, Ecuador; 10 Instituto de Biomedicina Universidad Católica de Santiago de Guayaquil and Centro de Investigación, Universidad Espíritu Santo, Guayaquil, Ecuador

**Keywords:** Medical genetics, Molecular medicine

## Abstract

An unusually high frequency of the lamellar ichthyosis *TGM1* mutation, c.1187G > A, has been observed in the Ecuadorian province of Manabí. Recently, the same mutation has been detected in a Galician patient (Northwest of Spain). By analyzing patterns of genetic variation around this mutation in Ecuadorian patients and population matched controls, we were able to estimate the age of c.1187G > A and the time to their most recent common ancestor (TMRCA) of c.1187G > A Ecuadorian carriers. While the estimated mutation age is 41 generations ago (~1,025 years ago [ya]), the TMRCA of Ecuadorian c.1187G > A carrier haplotypes dates to just 17 generations (~425 ya). Probabilistic-based inferences of local ancestry allowed us to infer a most likely European origin of a few (16% to 30%) Ecuadorian haplotypes carrying this mutation. In addition, inferences on demographic historical changes based on c.1187G > A Ecuadorian carrier haplotypes estimated an exponential population growth starting ~20 generations, compatible with a recent founder effect occurring in Manabí. Two main hypotheses can be considered for the origin of c.1187G > A: (*i*) the mutation could have arisen in Spain >1,000 ya (being Galicia the possible homeland) and then carried to Ecuador by Spaniards in colonial times ~400 ya, and (*ii*) two independent mutational events originated this mutation in Ecuador and Galicia. The geographic and cultural characteristics of Manabí could have favored a founder effect that explains the high prevalence of *TGM1* c.1187G > A in this region.

## Introduction

Inherited ichthyosis is a group of keratinization disorders characterized by generalized hyperkeratosis and scaling. These diseases are divided into two groups, syndromic ichthyosis and non-syndromic ichthyosis, according to the presence or absence of extracutaneous findings, respectively. Autosomal recessive congenital ichthyosis (ARCI) is a subgroup of non-syndromic ichthyosis, among which are included lamellar ichthyosis (LI; OMIM 242300), congenital ichthyosiform erythroderma (CIE; OMIM 242100) and harlequin ichthyosis (HI; OMIM 242500). There are also three other minor types of ARCI: self-healing collodion baby (SHCB), acral self-healing collodion baby (ASHCB) and bathing suit ichthyosis (BSI)^1^.

Lamellar ichthyosis is the most common type of ARCI; some of its clinical manifestations include: generalized scaling, ectropion, eclabium, collodion membrane at birth and palmoplantar keratoderma^2^. Although epidemiological data of LI is limited, its estimated prevalence ranges from 1:200,000 to 1:300,000 cases^3^.

*TGM1*^4^, is the most commonly mutated gene in LI. It encodes the transglutaminase 1 enzyme that is involved in the formation of the cornified cell envelope of the epidermis^2^.

A few founder mutations have been described in the *TGM1* gene. Pigg *et al.*^5^ demonstrated the existence of founder effects of a recurrent *TGM1* mutation, c.2526A > G, in Norwegian population. In Galicia (Northwestern Spain), three founder mutations, c.2278C > T, c.1223_1227delACAC and c.984 + 1G > A, account for the majority of all *TGM1* mutations identified in this Spanish region^6^.

Manabí is a province located in the northwest coast of Ecuador where a high prevalence of ARCI exists. Little attention was given to this particular isolated population until the foundation of Miss Ecuador organization (known as the “Club de la Ictiosis”) that has provided professional assistance and medicines free of charge to affected people. A total of 27 cases of ARCI in Manabí were identified by the “Club de la Ictiosis” in a population of 1,369,780 inhabitants (http://www.ecuadorencifras.gob.ec/censo-de-poblacion-y-vivienda); making an estimated prevalence of about 1:50,000 cases. Despite the high frequency of affected people with ARCI, only one genetic study has been conducted in these patients so far^7^. In the study by Zambrano *et al*.^7^, an exploratory genetic analysis on eleven ARCI patients from this relatively isolated region of Ecuador was performed; their results showed the existence of a highly prevalent mutation in *TGM1*, c.1187G > A (p.Arg396His), in seven homozygous and two heterozygous cases.

Given this background, and with the aim of shedding light in the most prevalent ARCI mutations in Manabí, we have now analyzed 19 patients from this region and their relatives. The c.1187G > A was identified in 11 patients, representing 55% of the mutant alleles in this sample. Interestingly, we have previously identified this same mutation in a Galician family with LI^8^.

Here we hypothesize that the high prevalence observed for c.1187G > A in Manabí is a consequence of a historical founder effect. Therefore, the aims of the present study are: (1) to analyze whether all c.1187G > A mutation carriers share a common haplotype which could signal a common ancestral origin, if so, (2) to estimate the time to their most recent common ancestor (TMRCA) by using different statistical approaches, and (3) to estimate the age of this mutation using different demographic parameters. Finally, with this information in mind, it might be possible to investigate whether the Ecuadorian mutation is original from America, or alternatively, if it could have been originated in Europe and then carried to America by Spanish colonizers, considering the recent historical connection between both populations.

## Material and Methods

### Sample collection

We studied a total of 11 Ecuadorian patients, from the 19 Ecuadorian ARCI patients analyzed in our service, carriers of the c.1187G > A mutation. These 11 affected individuals belonged to 7 apparently unrelated families (documented for at least three generations). They were all originally from the coastal province of Manabí in Ecuador (Table 1).

**Table 1:** Geographic origin and mutations of the Ecuadorian patients studied diagnosed with Lamellar Ichthyosis. Hom: homozygous.

Families	Family ID	Sample ID	Mut 1	Mut 2	Protein 1	Protein 2	Location
Family 1	1 A	III:2	c.1187G > A	c.2278 C > T	p.Arg396His	p.Arg760*	Jaramijó, Manabí
Family 2	5E	III:1	c.1187G > A (hom)	—	p.Arg396His (hom)	—	Jipijapa, Manabí
Family 3	11E	IV:1	c.1187G > A (hom)	—	p.Arg396His (hom)	—	Manta, Manabí
Family 4	24E	III:6	c.1187G > A	c.1192 G > T	p.Arg396His	p.Val398Phe	San Mateo, Manabí
Family 5	25E	IV:1	c.1187G > A (hom)	—	p.Arg396His (hom)	—	Manta, Manabí
Family 6	27E	III:3	c.1187G > A (hom)	—	p.Arg396His (hom)	—	Puerto López, Manabí
Family 7	17E	IV:3	c.1187G > A (hom)	—	p.Arg396His (hom)	—	San Lorenzo, Manabí
Family 7	17E	IV:5	c.1187G > A (hom)	—	p.Arg396His (hom)	—	San Lorenzo, Manabí
Family 7	17E	V:1	c.1187G > A (hom)	—	p.Arg396His (hom)	—	Manabí
Family 7	17E	IV:24	c.1187G > A (hom)	—	p.Arg396His (hom)	—	Manabí
Family 7	17E	IV:29	c.1187G > A (hom)	—	p.Arg396His (hom)	—	Manabí

A medical history, clinical photos and pedigrees reconstructed for at least three generations were available. Pedigree information is provided in Figs S1–S7.

STR (Short Tandem Repeat) genotyping (see below) used for the analysis of local ancestry was carried out on 11 Ecuadorian and one Galician (previously identified by our group^8^) c.1187G > A carriers. The reference populations for this analysis were: (*i*) 73 non-affected Ecuadorian citizens (146 haplotypes), which included 45 unrelated individuals from the general population and 28 unrelated individuals from 14 trios (only the haplotypic data of the parents was considered) plus 12 non-carrier haplotypes from our seven unrelated Ecuadorian families; and (*ii*) European controls represented by 100 Galician healthy individuals (200 haplotypes) plus the non-carrier haplotype of the unique heterozygous Galician case.

SNPs (Single Nucleotide Polymorphisms) used to estimate global genome ancestry, were genotyped (see below) in nine Ecuadorian c.1187G > A carriers (patients 1 A, 5E, 25E, 27E, 17E.IV:3, 17E.IV:5, 17E.V:1, 11E and 24E). As reference for the Spanish ancestry, we used the SNP genotypic information of 79 unrelated Spanish ARCI patients previously analyzed in our laboratory (ESP), including the Galician c.1187G > A carrier.

We obtained written informed consent for all participants prior the research, which includes consent for publication of individual data. All procedures followed were in accordance with an ethical approval by a Regional Institutional Review Board that complies with the Declaration of Helsinki.

### STR haplotyping

Ten polymorphic microsatellite markers (STR) were used to study the 14q12 locus spanning a 12 Mb region flanking the *TGM1* gene. Microsatellite data was obtained from the study reported by Fachal *et al*.^6^.

Forward PCR primers were labeled with either FAM or HEX fluorescent dyes (Sigma-Genosys Ltd. Cambridgeshire, UK). The amplification products were separated by capillary electrophoresis on an ABI3730xL (Applied Biosystems) sequencer and analyzed with GeneMapper v4 Software. All reactions were performed according to the manufacturer’s protocols.

PHASE v2.1^9^ software was used for haplotype reconstruction of Galician and Ecuadorian controls, which benefits from few available trios data. Patients haplotypes were phased by manual inspection using data available from trio-families and taking advantage of the fact that all of them share a common recent mutation.

### TMRCA and mutation age estimation

Estimates of both the TMRCA and mutation age are usually a very complex task. To the best of our knowledge there is no single method strongly recommended, although models with different approaches are widely used. Therefore, the TMRCA was estimated using two approaches based on the analysis of linkage disequilibrium (LD) patterns around the c.1187G > A mutation. The first approach is known as the single marker method, which we applied by using four different algorithms proposed by Bergman *et al*.^10^, Labuda *et al*.^11^, Risch *et al*.^12^ and Lander and Botstein *et al*.^13^. These algorithms assume that the mutation will be in LD with nearby marker alleles at polymorphic loci, and decay of this LD over time (through recombination) provides information about the TMRCA. Estimates were calculated individually for 9 markers. D14S264 was not taken into consideration due to its complete LD with the targeted mutation. The marker D14S1043 was not evaluated by the Bergman’s estimator because the founder allele was more common in the control than in the diseased population. In the case of Risch’s algorithm we also excluded D14S581, D14S64, D14S1042 markers due to the frequency of their alleles, which was the same as the most adjacent marker of the conserved region. The median was applied to the results derived from each single marker to obtain a unified estimate of the TMRCA. The physical distances were converted into centiMorgans (cM) assuming that 2.22 cM is equal to 1 Mb; this equivalence was calculated using the recombination data obtained from deCODE genetic map available on the UCSC database^14^. The Haldane map function^15^ was used for translation of map distances into recombination frequencies (See Table S1). Bergman and Labuda algorithms were corrected with the correction factor proposed by Labuda *et al*.^15^ (Table S1), that considers the population growth rate. The population growth rate, *r* = 0.1658, was obtained using demographic data of Ecuadorian population, historical data from 1495 onwards^16–18^, and assuming 25 years per generation.

The second approach used for estimating the TMRCA was the gamma method published by Gandolfo *et al*.^19^ based on the genetic length of ancestral haplotypes shared between individuals carrying the same mutation. The ancestral haplotypes are limited by the outermost markers which do not share the founder allele from each side of the mutation.

**Figure 1:**
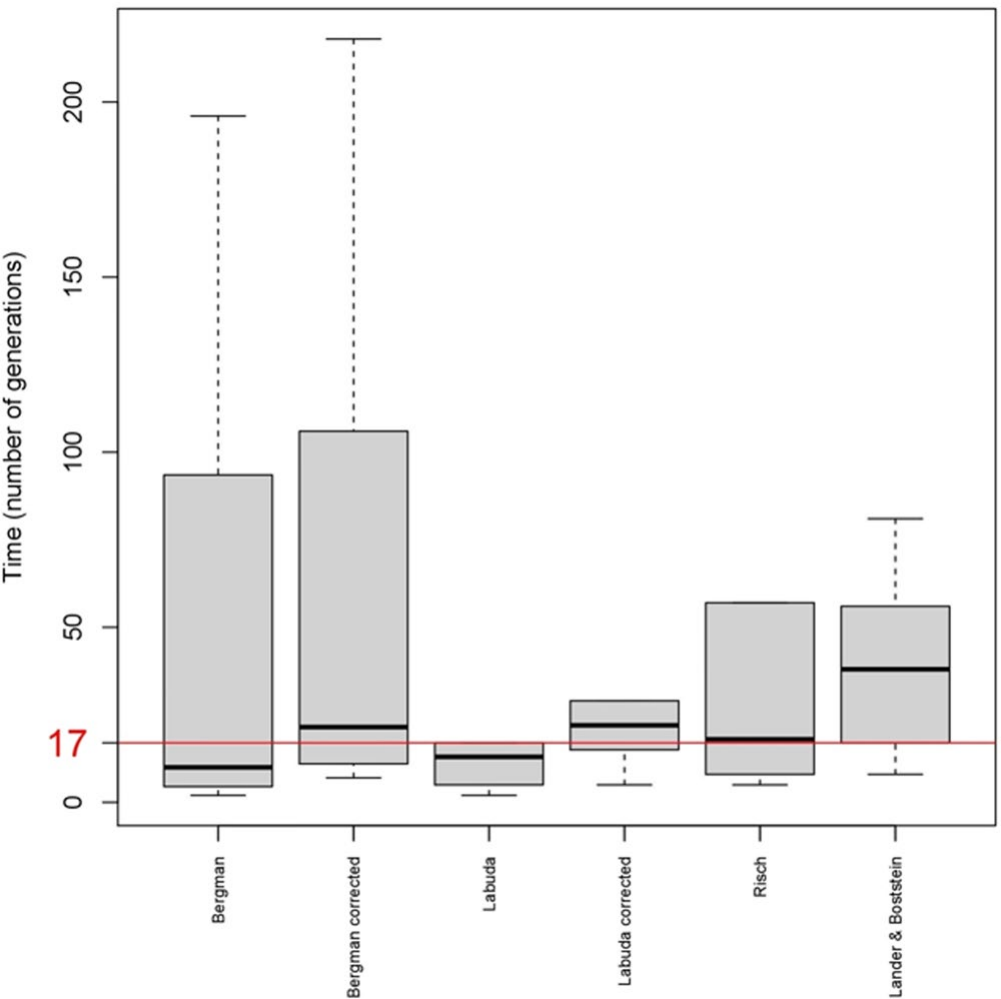
Boxplot of TMRCA estimations (years rounded to the nearest integer) using different methods. The red line represents the common estimation among all markers. Outliers have been removed from the plot.

There are some decisions and limiting factors for the estimation of the TMRCA, including: (a) choice of the founder allele, (b) the sample size (LD block reconstruction is sensitive to sample size; e.g. larger samples may have smaller core haplotypes), (c) haplotyping errors (e.g. influencing the observed allele frequencies), (d) the existing variation in recombination frequencies between different genetic maps and populations, (e) the fact that single marker estimators can only be used when the founder allele is more common in patients than in controls, and (f) the limited knowledge of reported demographic variation available in this Ecuadorian region along the history. Despite the limitations, our TMRCA estimates were consistent when using the different approaches (Table S1 and Fig. 1).

DMLE+^20^ software was used to calculate the number of generations since the mutation occurred. This method compares the LD of mutation carrier markers and unaffected control group markers. It also requires other genetic and demographic information, including map distances among marker loci, mutation location, population growth rate, and proportion of population sampled. The proportion of population sampled was calculated considering that the total number of patients suffering from ARCI in the province of Manabí was 27 (according to the numbers published by “Club de la Ictiosis”). The estimated prevalence is about 1:50,000 cases (see above). We have analyzed 19 of the 27 patients (~70%) and found that 9 out of the 19 cases carry the c.1187G > A mutation in homozygosis (~47%). We can therefore infer that ~13 patients (47% of 27) have this mutation in homozygous state. Following the Hardy-Weinberg principle, we estimate the frequency of this mutation in ~0.003. Although the presence of this mutation can affect the fitness of the carriers, it is reasonably safe to assume Hardy-Weinberg equilibrium giving that most of the mutations survived in heterozygotes. According to the ExAC database (http://exac.broadinstitute.org) the frequency of this variant in the general population is 2.5 × 10^−05^. Considering the estimated allele frequency in this population, and the present population size of Manabí, there are about 8,596 estimated c.1187G > A carrier chromosomes in this region of Ecuador. Thus, the proportion of the carrier chromosome sampled in the present study can be estimated as 20/8596, namely, 2.3 × 10^−03^.

### Genome ancestry analysis based on SNP data

To study the genomic variability in the Ecuadorian families we used marker data, comprised of 10,938 SNPs obtained from a custom 40 gene targeted resequencing panel previously used for the genetic diagnosis of our Ecuadorian cohort of ARCI patients. The SNPs showing an LD r^2^ > 0.5 were removed and a total of 2,661 SNP markers were used for the analysis. For further details see Table S2.

Apart from using the genotyping data of our nine Ecuadorian c.1187G > A carriers and Spanish controls, population data generated by the 1000 Genomes Project database^21^ (1000G) were also used as continental references for ancestry inference. We intersected our SNPs in the targeted resequencing panel with 1000G genotyping data using previously described procedures^22,23^. We selected three populations representing the expected main ancestral continental groups of Ecuador: IBS-1000G (Iberian Population in Spain), YRI-1000G (Yoruba in Ibadan, Nigeria) and PEL-1000G (Peruvians from Lima, Peru). In order to configure a more compact Native American cluster in the ancestry analysis, a total of 40 PEL-1000G samples with European ancestry >15% (data no shown) were removed from the analysis. A total of 2,661 SNPs and 492 individuals were finally used for ancestry analysis.

Multidimensional Scaling (MDS) was carried out on a matrix of pairwise individual identity-by-state (IBS) values with the aim of exploring clusters of genetic variation in the population sets analyzed. MDS was performed using the function *cmdscale* (library stats) from R^24^. Using the same filtered panel of SNPs, we estimated maximum likelihood individual ancestries using ADMIXTURE^25^.

### Estimation of local ancestry of c.1187G > A haplotypes

Analysis of local ancestry was carried out on Ecuadorian (*n* = 20) and Galician (*n* = 1) c.1187G > A carrier chromosomes. As reference populations for classification, we used the Ecuadorian (*n* = 158), and the Galician (*n* = 201) control haplotypes. Haplotypes of carrier patients, including both Ecuadorian and Galician, are represented in Table 2, while control haplotypes are shown in Table S3.

**Table 2:** Phased haplotypes of the eleven patients who carry the *TGM1*: c.1187G > A mutation for the 10 STR markers. Some patients are homozygous carriers (Hom; two haplotypes represented) and some are heterozygous carriers (Het; one haplotype represented.

Patient	Haplotypes ID	STR MARKERS										
		D14S72	D14S1043	D14S742	D14S581	D14S64	Mutation	D14S264	D14S1032	D14S275	D14S1042	D14S1060
FAM 1 A (III:2)	h1 (ECU/ECU)	2	5	3	3	8	HET	9	9	4	4	6
FAM 5E (III:1)	h2 (ECU/ECU)	2	5	3	3	8	HOM	9	9	4	4	6
	h3 (ECU/ECU)	2	5	3	3	8		9	9	4	4	6
FAM 25E (IV:1)	h4 (ECU/ECU)	2	5	4	1	8	HOM	9	9	4	4	6
	h5 (ECU/ECU)	2	5	4	1	8		9	9	4	4	6
FAM 27E (III:3)	h6 (ECU/ECU)	7	5	1	3	8	HOM	9	9	4	4	6
	h7 (ECU/ECU)	7	5	3	3	8		9	9	4	4	6
FAM 17E (IV:3)	h8 (ECU/ECU)	7	5	3	2	5	HOM	9	9	8	4	10
	h9 (ECU/ECU)	2	5	3	3	8		9	9	4	4	6
FAM 17E (IV:5)	h10 (ECU/ECU)	7	2	2	2	5	HOM	9	7	8	10	4
	h11 (ECU/ECU)	7	5	3	3	8		9	9	3	4	6
FAM 17E (V:1)	h12 (GAL/GAL)	7	4	3	2	5	HOM	9	8	4	4	4
	h13 (GAL/GAL)	8	4	4	2	5		9	9	4	4	6
FAM 17E (IV:29)	h14 (GAL/GAL)	8	6	3	2	5	HOM	9	9	4	4	5
	h15 (?/GAL)	8	6	3	2	5		9	9	4	4	6
FAM 17E (IV:24)	h16 (ECU/GAL)	7	2	3	2	5	HOM	9	6	8	4	6
	h17 (ECU/ECU)	8	5	3	2	5		9	9	4	4	6
FAM 11E (IV:1)	h18 (ECU/GAL)	7	2	3	2	5	HOM	9	6	8	6	6
	h19 (ECU/ECU)	7	5	3	3	8		9	9	4	4	6
FAM 24E (III:6)	h20 (ECU/ECU)	7	5	3	2	5	HET	9	7	4	4	4
Galician haplotype	–	7	5	3	2	4	HET	6	8	6	8	8

The Galician c.1187G > A carrier haplotype is also included. The inferred ancestral origin of the Ecuadorian haplotypes (see text) is indicated in column “Haplotype ID” in brackets (PCA-QDA/SVM classification); GAL = most likely Galician origin; ECU = most likely Ecuadorian origin. It was not possible to infer the origin of h15 using PCA-QDA because the higher probability of classification was below to 0.75.

The most likely geographic origin of STRs haplotypes (providing information on local ancestry of the mutation) was estimated using two different procedures. The first method is the algorithm described by Egeland *et al*.^26^ that is based on an initial principal component analysis (PCA; function *prcomp*, library stats) combined with quadratic discriminant analysis (QD; functions *qda* and *predict*, from libraries MASS and stats respectively) implemented in R (http://www.r-project.org). Only haplotypes with at least 0.75 highest probability of classification were considered; one haplotype could not be classified according to this criterion (Table 2, h15). The number of principal components for classification in the PCA step was decided by following the criterion of retaining a cumulative variance of at least 80%. This method was successfully used to estimate the most likely geographic origin of uniparental haplotypes^27^.

A leave-one-out cross-validation (LOOCV) procedure was followed to eliminate non-Native American haplotypes from the Ecuadorian reference populations.

The second method for classification was the support vector machine (SVM) procedure^28^.

### Effective population size through time

Extended Bayesian Skyline Plots (EBSPs) were obtained using BEAST v.2.^29^. Ecuadorian cases and controls haplotype data were analyzed in order to study the changes in the effective Ecuadorian population size (*N*_e_) through time. The site models, the clock models, and partition trees of the different loci were linked. For the substitution model, we specified proportional rates, linear mutation bias and a one phase model according to the data published by Sainudiin *et al*.^30^. We used a fixed mutation rate of 10^−3^ substitutions/microsatellite/generation based on the mutation rate estimate of Sun *et al*.^31^. Default priors were used for model parameters and statistics, except that the demographic population mean was set to uniform, with an initial value of 40,000 and lower and upper bounds of 30,000 and 50,000; respectively. This decision was based on the results of an exploratory BEAST run with constant size tree prior using control samples as previously described by Teske *et al*.^32^. Each run was repeated at least twice with the same settings to ensure that searches converged on similar values.

## Results

### Haplotypes of c.1187G > A carrier families

We analyzed twenty haplotypes of eleven patients from seven different *TGM1*: c.1187G > A carrier families. Two out of the eleven patients were heterozygous while the rest were homozygous for this mutation. Different haplotype frequencies were found in control’s and patient’s chromosomes. All c.1187G > A carrier families shared a common marker, D14S264, although the rest of markers did not all present the same allele, a 8.7 Mb prevalent core haplotype (9-9-4-4-6) was identified for five markers D14S264, D14S1032, D14S275, D14S1042 and D14S1060 on twelve out of twenty chromosomes (60%). This core haplotype was not found in any of the control chromosomes. A visual inspection of the haplotypes represented in Table 2 allow us to roughly differentiate two main groups by focusing in the two 5′-STRs that are close to the mutation: one group share the D14S581-D14S64 haplotype 2–5, while the other group share the haplotype 3–8; both haplotypes accounting for 18 out of 20 haplotypes.

### TMRCA and age estimation of c.1187G > A

A deep analysis of the patterns of LD around a mutation of interest can provide information on its age as well as details on the historical demography underlying these genetic patterns. Results derived from the application of different TMRCA estimators are shown in Fig. 1 and Table S1. The TMRCA was estimated to be 10 generations by Bergman’s estimator within an interquartile range (IQR) of 5 to 10 generations. When applying the correction proposed by Labuda the estimated time was 22 generations within an IQR of 11–22. The rest of the methods produced narrower intervals. Thus, Labuda’s algorithm gave a result of 13 generations ranging from 5 to 13 whereas the number of generations estimated was higher when considering Labuda’s correction factor, 22 generations within an IQR of 15 to 22. Risch and Lander and Botstein algorithms dated the TMRCA to 18 (IQR 8–18) and 38 generations (IQR 17–38), respectively.

According to the gamma method, the TMRCA was quite close to the previous estimators, approximately 27 generations (95% CI: 20–37). The variation of the median is represented in a boxplot for each single marker method in Fig. 1. All TMRCA estimates overlap at 17 generations, this value is also close to the median of the different estimates.

The *TGM1* c.1187G > A mutation, the most common cause of LI in our Ecuadorian cohort of patients, was estimated by DMLE to have arisen 41 generations ago (95% CI: 32–58). Therefore, assuming 25 years per generation, this estimate can be translated into ~1,025 years (Table S1).

### Genome ancestry of c.1187G > A carriers

**Figure 2:**
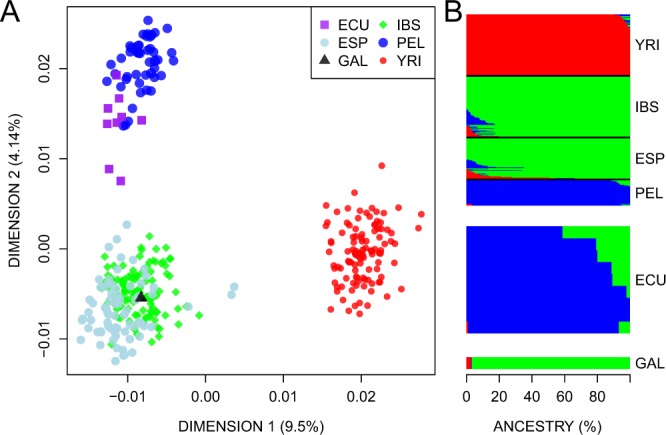
(**A**) MDS using autosomal SNPs of population samples from Ecuador, Spain, Peru, Nigeria and China. Population codes are as follows: ECU = Ecuadorian c.1187G > A carrier patients (present study), ESP = Spanish ARCI cases without the *TGM1* c.1187G > A mutation (present study), GAL = Galician (Spanish) case with the *TGM1* c.1187G > A mutation (present study), IBS = Iberian population in Spain, PEL = Peruvians from Lima (Peru), YRI = Yoruba in Ibadan (Nigeria) (all from 1000G^21^). (**B**) Barplot indicating ancestral memberships for the reference populations in 1000G and the SNP profiles of Ecuadorian carriers and Spanish ARCI patients.

The MDS plot of Fig. 2A aims at exploring the genome continental ancestry of c.1187G > A carriers. Dimension 1 separates the African profiles from the rest of the continental haplotypes, while Dimension 2 separates the Europeans (represented by IBS-1000G and our controls) from the Native American pool (represented by PEL-1000G). Two Ecuadorian profiles occupy a position that is closely related to the Spanish profiles (IBS and ESP), while the unique Galician c.1187G > A-haplotype is clearly integrated in the Spanish cluster.

Admixture patterns of Ecuadorian and Spanish SNP profiles agree well with MDS results (Fig. 2B). The main ancestral membership of Ecuadorian samples is shared with Peruvians (the closest surrogated Native American population to Ecuador in 1000G), indicating their main Native American ancestry (87.3% on average). However, almost all of them share ancestry with Europeans (12.6% on average; the two highest values being 41% and 21%). Finally, the Galician case fits fully within the European ancestry (Fig. 2B).

### Local ancestry of c.1187G > A STR-haplotypes

**Figure 3:**
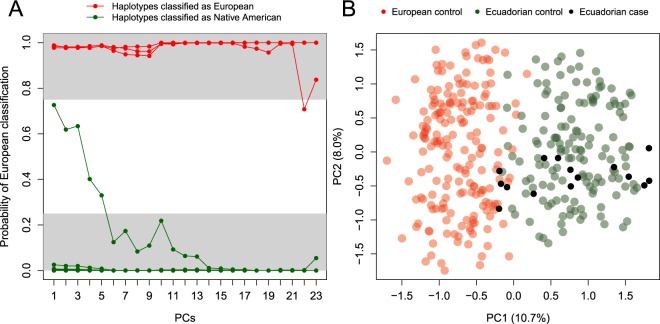
Most likely geographical origin of the c.1187 A > G Ecuadorian STR-haplotypes. (**A**) PCA-QDA probabilistic classification of Ecuadorian c.1187A > G haplotypes as a function of 23 accumulated principal components. (**B**) PCA of the first and second principal components showing haplotypes from cases and control subjects from Ecuador and Europe (see Material and Methods).

The PCA-QDA classification algorithm aimed at inferring the most likely origin of c.1187G > A STR-haplotypes (local ancestry) allowed to classify 19 out of 20 Ecuadorian haplotypes. This analysis indicated that ~16% (3 out of 19) of the Ecuadorian c.1187G > A haplotypes are classified as European (Fig. 3A; Table 2).

Figure 3B shows the first two principal components out of the 23 used by the classification algorithm (Fig. 3A). By using these two components, this PCA plot already indicates the existence of two main clusters that group the Ecuadorian control haplotypes on one side of PC1, and the European ones on the other side; while most of the patient haplotypes fall in the Ecuadorian side, a few haplotypes plot more closely related to the European pole.

In addition, the use of SVM for the classification of Ecuadorian cases’ haplotypes raises to 30% the proportion of the Ecuadorian cases’ haplotypes classified as European. Moreover, the three Ecuadorian haplotypes classified as European using PCA-QDA were consistently classified as European using SVM (Table 2).

### Demographic inferences of STR-haplotypes

Demographic patterns were examined independently for haplotypes observed in Ecuadorian cases and controls. The EBSP showed totally different patterns in these two groups.

**Figure 4:**
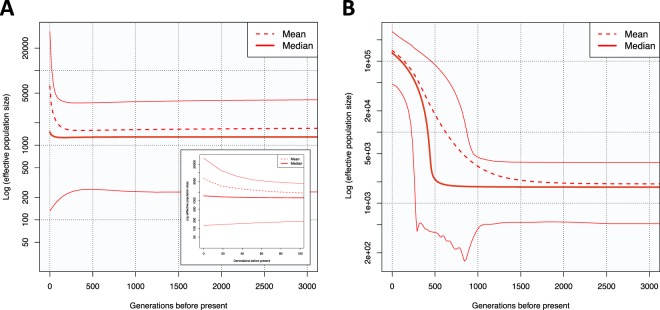
Extended Bayesian Skyline Plot (EBSP) of Ecuadorian population size through time. Mean, median and 95% HPD (Highest Posterior Density) population size are shown. (**A**) EBSP of Ecuadorian patients’ haplotypes with the *TGM1*: c.1187A > G mutation, and (**B**) EBSP of Ecuadorian controls’ haplotypes.

The analysis indicates that Ecuadorian haplotypes experienced a significant population growth in the last 20 generations (~500 years; Fig. 4A), while the Ecuadorian controls signal the existence of an important population expansion occurring ~400 generations ago (10,000 ya; Fig. 4B). Virtually the same time estimate was obtained when using equal number of haplotypes in cases and controls (data not shown).

## Discussion

The main aim of the present study was to investigate the origin of the *TGM1* mutation, c.1187G > A, which is found in most of the ARCI families living in an isolated coastal region of Ecuador, the province of Manabí. This same mutation was previously reported by Farasat *et al*.^33^ in an EEUU cohort which included ‘Hispanic’ descendants, one French case of ASHCB^31^ and in one Omani family^32^. In addition, we have recently found this mutation in a Galician patient^8^. The important demographic impact of Spaniards in Ecuador in historical and present times raises the question of a common Spanish origin for all the c.1187G > A carriers observed in Ecuador.

The demographic and genetic characteristics observed in Ecuadorian patients point to a historical founder effect for the c.1187G > A mutation. On the one hand, Manabí has complex orographic characteristics (e.g. steep mountains and deep valleys) that facilitate isolation and consanguinity between families. On the other hand, the data presented in this study derived from the analysis of STR markers flanking the *TGM1* gene in Ecuadorian c.1187G > A carriers, revealed the existence of a 8.7 Mb major haplotype, suggesting that these patients have a common origin.

To further investigate the plausible origins of this mutation, we carried out different analysis, including the estimation of their TMRCA and mutation age.

TMRCA estimate for c.1187G > A haplotypes was in the range of 10–38 generations which dates the common ancestor’s first appearance in an approximate interval of 250–950 ya. The consensus TMRCA estimate obtained from the different methods was 17 generations, which is compatible with a local founder who lived approximately 425 ya, and coincides with the time of the European conquest of America.

Nonetheless, according to DMLE results, the mutation may have arisen about 1,025 ya (41 generations). The PCA locates a number of the Ecuadorians patients´ haplotypes into the Spanish ancestry cluster suggesting that the haplotypic pool of Ecuador contains a mixture of European and Native American ancestry (Fig. 3). In addition, the classification algorithm roughly estimates the existing admixture in 16–30% European *versus* 84–70% Native American; these figures are well below the estimates obtained for other haplotypic markers in Ecuador^27^.

**Figure 5:**
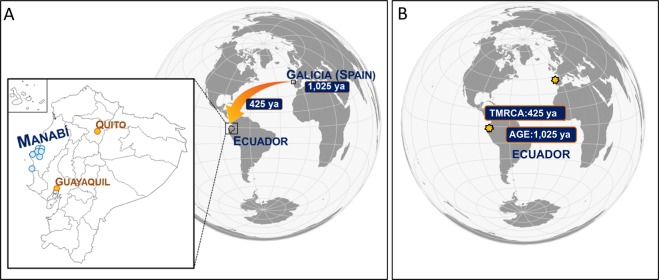
Schematic representation of the two proposed scenarios that could explain the origin of the *TGM1*: c.1187A > G mutation. (**A**) This model suggests the Galician (Spanish) origin of the c.1187A > G mutation found in Ecuadorian families. The geographic location of the Ecuadorian families (Manabí province) is indicated as circles. Largest Ecuadorian cities were also marked as reference points. (**B**) This model represents the dual origin, Ecuadorian and European, of the c.1187A > G mutation as a result of two independent mutational events.

Taking all the results together, we conceive two possible scenarios for the origin of the c.1187G > A mutation (Fig. 5). The first scenario would consider the origin of c.1187G > A somewhere in Europe ~1,025 ya. According to this hypothesis, all the haplotypes in Spain and Ecuador would have a common European ancestral origin (Fig. 5A). Recombination around the mutation in combination with former haplotypes which existed in Manabí before the arrival of Europeans would explain why a proportion of Ecuadorian carrier haplotypes classify as Native American while other Ecuadorian haplotypes still remain evolutionarily more closely related to the European haplotype pool. The Spanish territory of Galicia (Northwestern corner of Spain) would be a good candidate region for the c.1187G > A Ecuadorian mutation due two main reasons: (a) recently, the same mutation was found in a Galician patient (of European ancestry)^8^, and (b) another *TGM1* mutation was also recently found in one of the Ecuadorian families^7^, c.2278 C > T, which was previously described to cause founder effects in the Galician population^6,8^. Furthermore, this Spanish region has recorded several other cases of founder effects related to other diseases^6,34–40^. Therefore, both founder mutations could have arisen in Galician founder chromosomes and been brought to Ecuador during the colonization period about 425 ya, fitting with the TMRCA for this mutation in Ecuador. This model would be further supported by the different patterns of population size changes estimated for Ecuadorian cases´ haplotypes and controls. The haplotypes observed in Ecuadorian patients have a signal indicating an important population growth ~20 generations ago, again, roughly fitting the arrival of Spaniards to Ecuador. The colonial process favored the demographic growth of Spanish settlers, and this might have favored the rise of prevalence of c.1187G > A-in Manabí European carrier haplotypes. Concomitantly, the Native American variation was gradually disappearing or assimilated by Europeans^40^. Consistently, the haplotypes observed in Ecuadorian controls recorded an important population expansion dating back to the South American settlement by Upper Paleolithic hunter-gatherers from North and Mesoamerica 10,000 ya (400 generations approximately)^41–45^.

The second possible scenario would involve a dual independent origin of c.1187G > A in Galicia (with a European ancestral background) and Ecuador (with a Native American ancestral background); Fig. 5B. In Ecuador, this mutation would have arisen about 1,000 ya, with a TMRCA of about 400 ya. In Galicia there are not enough data to estimate the supposed independent origin of this mutation. The main finding favoring this scenario is that the largest proportion of carrier haplotypes in Ecuador are of most likely Ecuadorian origin. To explain this hypothesis, we should assume that a number of recombinational events could have driven the local ancestry of some Ecuadorian carrying haplotypes (16%) towards a main European ancestry.

Among the limitations of the present study, it is worth to mention that ancestral inference of STR-haplotypes carrying the *TGM1* c.1187G > A mutation was computed using control Ecuadorian STR-haplotypes masked for European ancestry; some non-Native American haplotype could have remained in this masked training set which could bias the estimation of ancestry of Ecuadorian *TGM1* c.1187G > A haplotypes. It is important to note however that classification probabilities are particularly high for the haplotypes that received assignation of European ancestry.

In addition, caution is needed considering that the methods used are sensible to isolated populations with high consanguinity and high allelic diversity. Moreover, the haplotype architecture we observed in the samples analyzed is complex due to a compounded interplay of evolutionary forces (e.g. recombination, mutation, genetic drift, population history, etc). Therefore, further investigations with larger samples could help to disentangle this complex population scenario.

Although reasonable doubts remain regarding the biogeographical origin of the c.1187G > A mutation, it seems most likely that the Ecuadorian c.1187 A > G carrier patients are all descendants of a common ancestor who lived >1,000 ya. Furthermore, the geographical and cultural characteristics of Manabí would favor a founder effect that could explain the high prevalence of this mutation in this isolated region of Ecuador.

## Supplementary information

Supplementary data Final

Table S1

Table S2

Table S3
