# Supplementary material for: Biogeographical origin and timing of the founder ichthyosis TGM1 c.1187G > A mutation in an isolated Ecuadorian population
Source: Sci Rep. 2019 May 9;9:7175. doi: 10.1038/s41598-019-43133-6 (PMC6509209; doi:10.1038/s41598-019-43133-6)
Supplement: Supplementary file 1 — Supplementary data Final [file 41598_2019_43133_MOESM1_ESM.docx]

*Supplementary data*

**Biogeographical origin and timing of the founder ichthyosis *TGM1* c.1187G>A mutation in an isolated Ecuadorian population**

Uxia Saraiva Esperón-Moldes^1,2^, Jacobo Pardo-Seco^2,3^, Martha Montalván-Suárez^4^, Laura Fachal^1^, Manuel Ginarte^5^, Laura Rodríguez-Pazos^6^, Alberto Gómez-Carballa^3^, María Fernanda Moscoso^7^, Nora Ugalde-Noritz^8^, Andrés Ordóñez-Ugalde^1,7,8^, Daniel Tettamanti-Miranda^9^, Juan Carlos Ruiz^10^, Antonio Salas^2,3,#,^*, Ana Vega^1,#,^*

This file is composed of 7 figures and 3 tables attached as a separate XLSX file

**Figure S1.** Pedigree of family 1E.

**Figure S2**. Pedigree of family 5E.

**Figure S3.** Pedigree of family 11E.

**Figure S4**. Pedigree of family 17E.

**Figure S5.** Pedigree of family 24E.

**Figure S6.** Pedigree of family 25E.

**Figure S7.** Pedigree of family 27E.

**Table S1**. TMRCA and mutation age estimations. Detailed information of each marker including: distance to the mutation in Mb, estimated recombination fraction, founder allele in cases and controls. TMRCA calculations in generations for each marker and Labuda´s correction also in generations. The botton row shows the median of the TMRCA age estimation for each estimator and the interquartile range (IQR), Gamma method and DMLE results are given in number of generations with a confidence interval of 95%. θ: recombination fraction according to Haldane mapping function, PD: frequency of the founder allele in the normal population, PN: frequency of the founder allele in the disease population.

**Table S2**. Markers used for the ancestral study, including their chromosomal location, the number of markers before and after LD (Linkage disequilibrium) filtering.

**Table S3**. Phased haplotypes of the control population (Ecuador and Galicia) for the 10 STR markers.


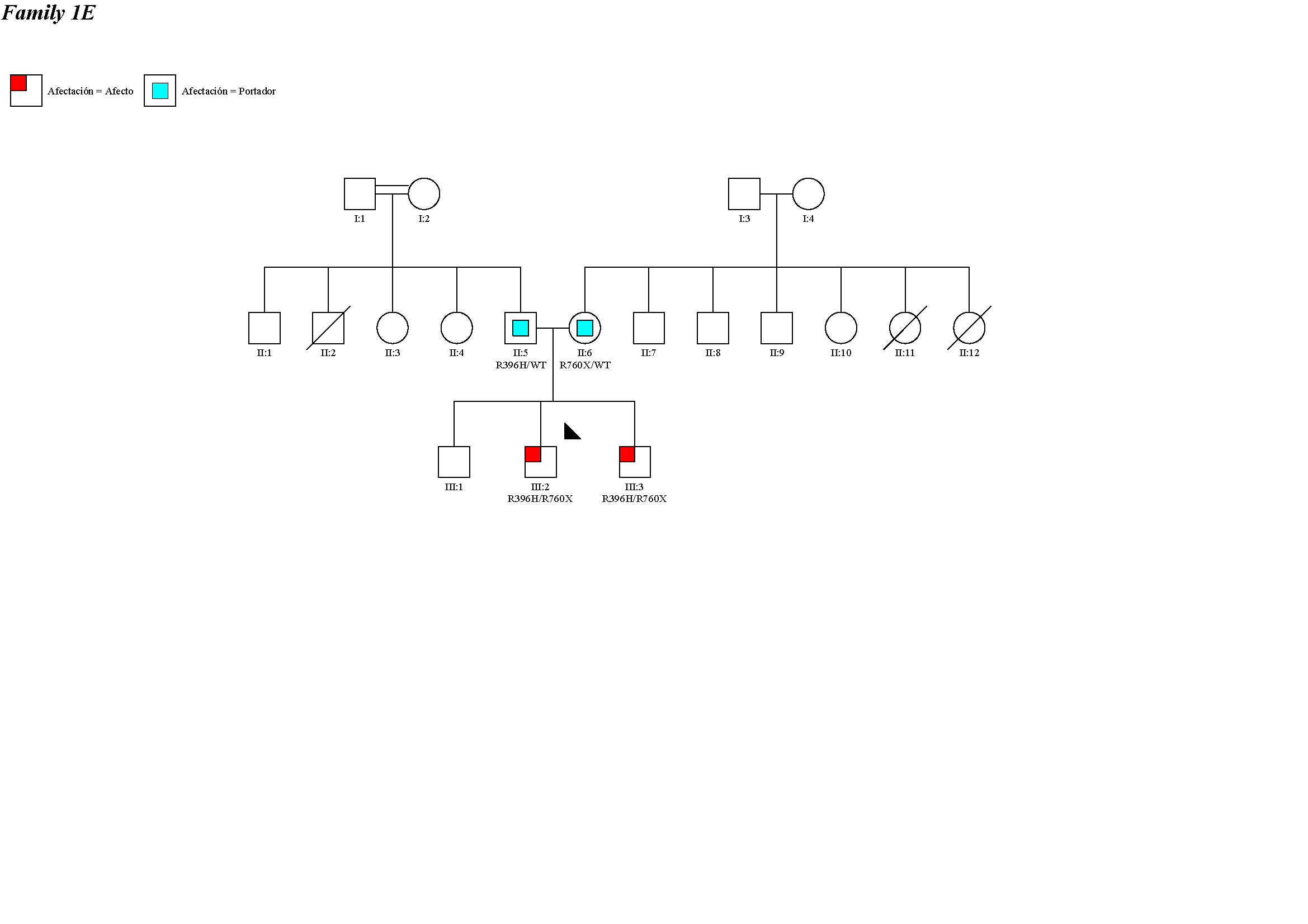
Figure S1. **Pedigree of family 1E.** Affected subjects and carriers are denoted with red and blue squares, respectively. The proband is marked with an upper left arrow.

Figure S2. **Pedigree of family 5E.** Affected subjects and carriers are denoted with red and blue squares, respectively. The proband is marked with an upper left arrow.


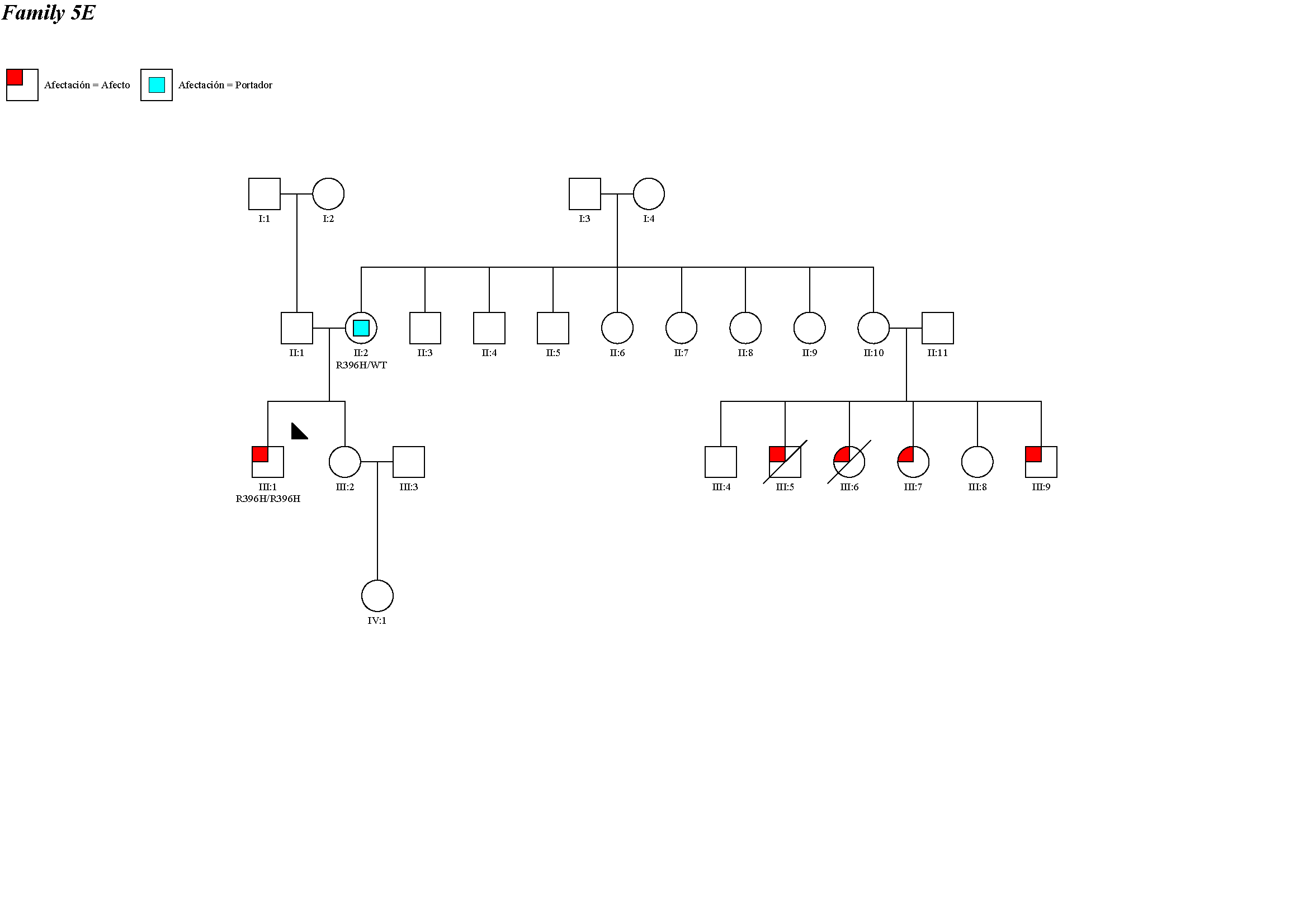


Figure S3. **Pedigree of family 11E.** Affected subjects and carriers are denoted with red and blue squares, respectively. The proband is marked with an upper left arrow.


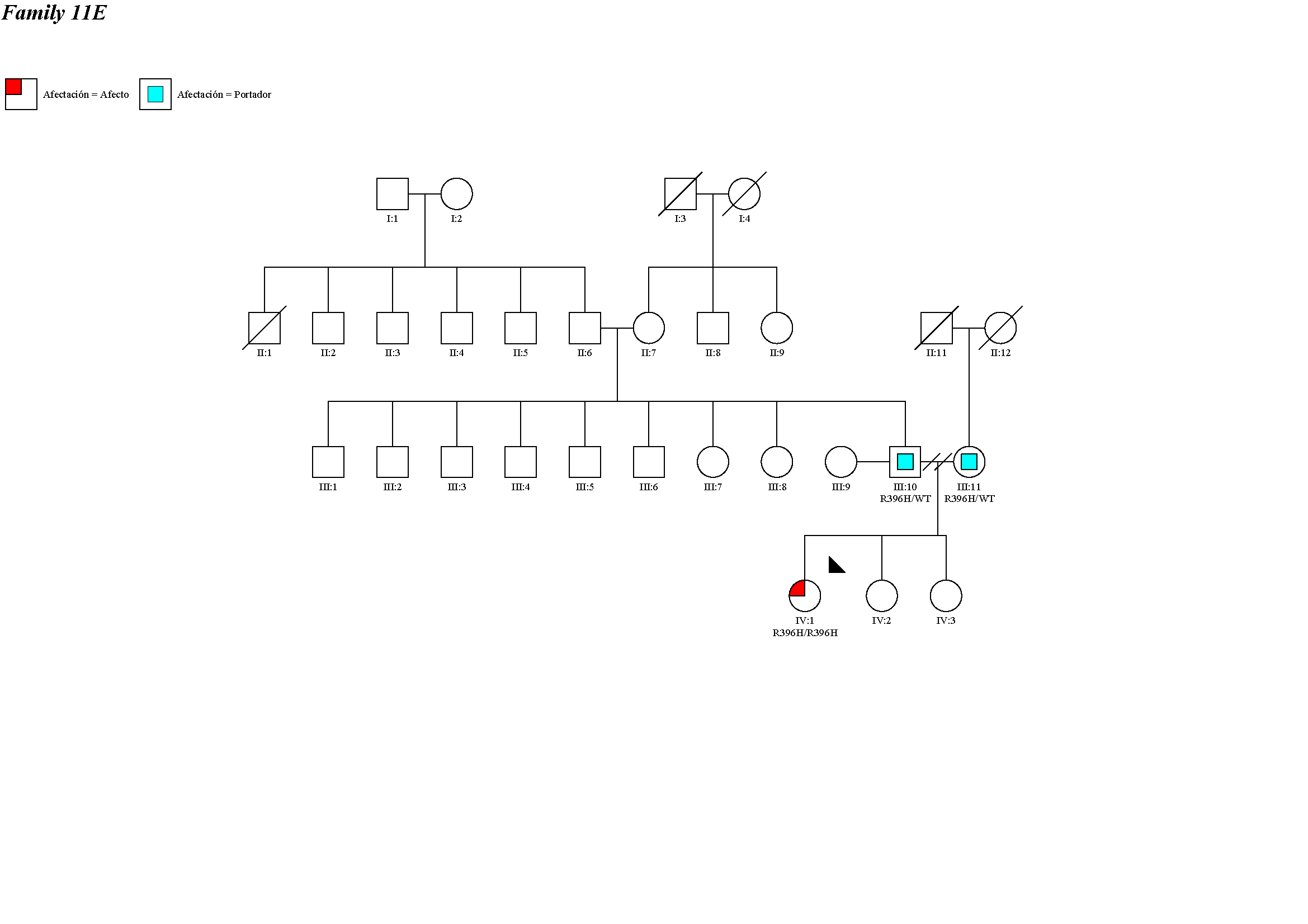


Figure S4. **Pedigree of family 17E.** Affected subjects and carriers are denoted with red and blue squares, respectively. The proband is marked with an upper left arrow.


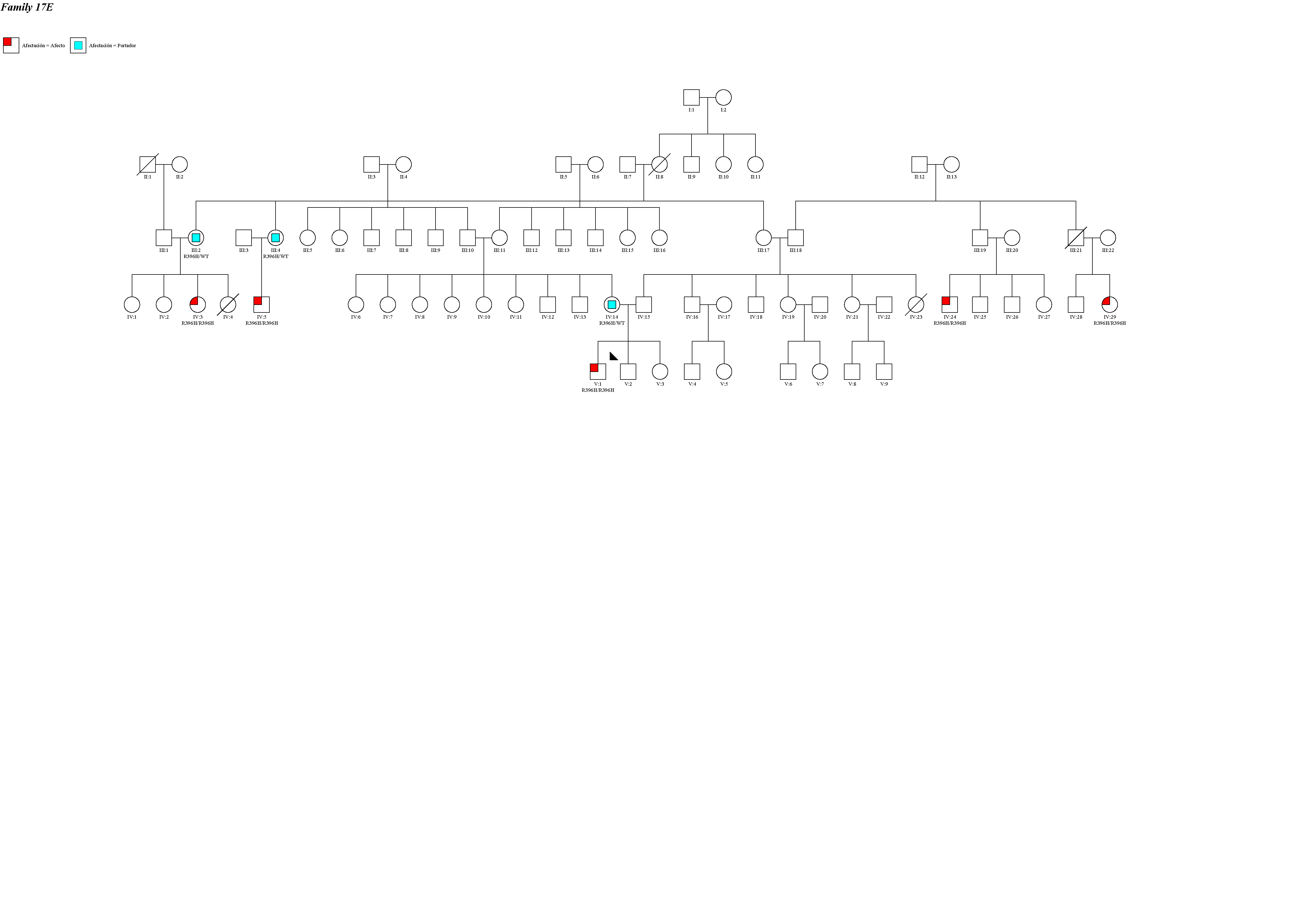


Figure S5. **Pedigree of family 24E.** Affected subjects and carriers are denoted with red and blue squares, respectively. The proband is marked with an upper left arrow.


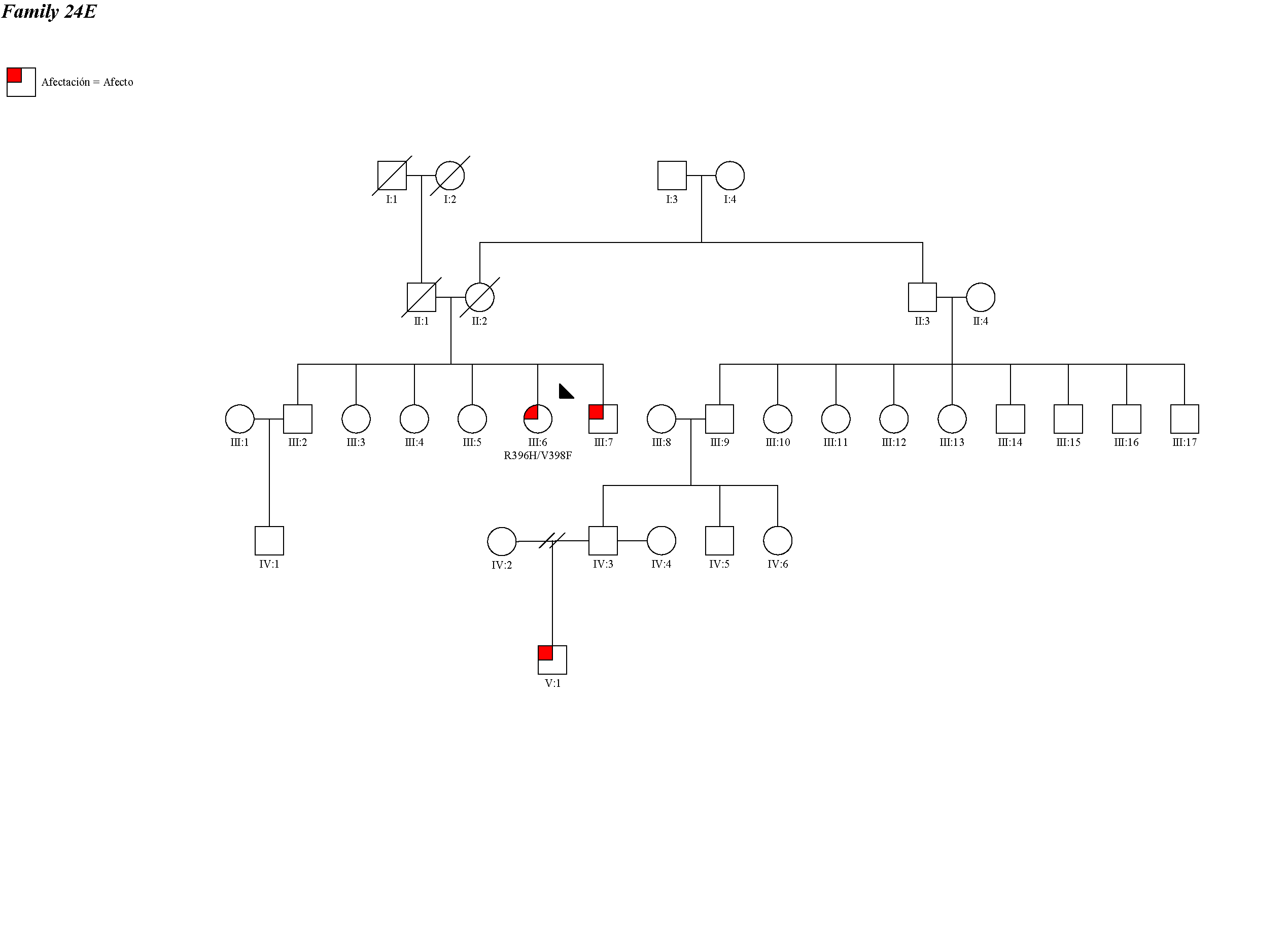


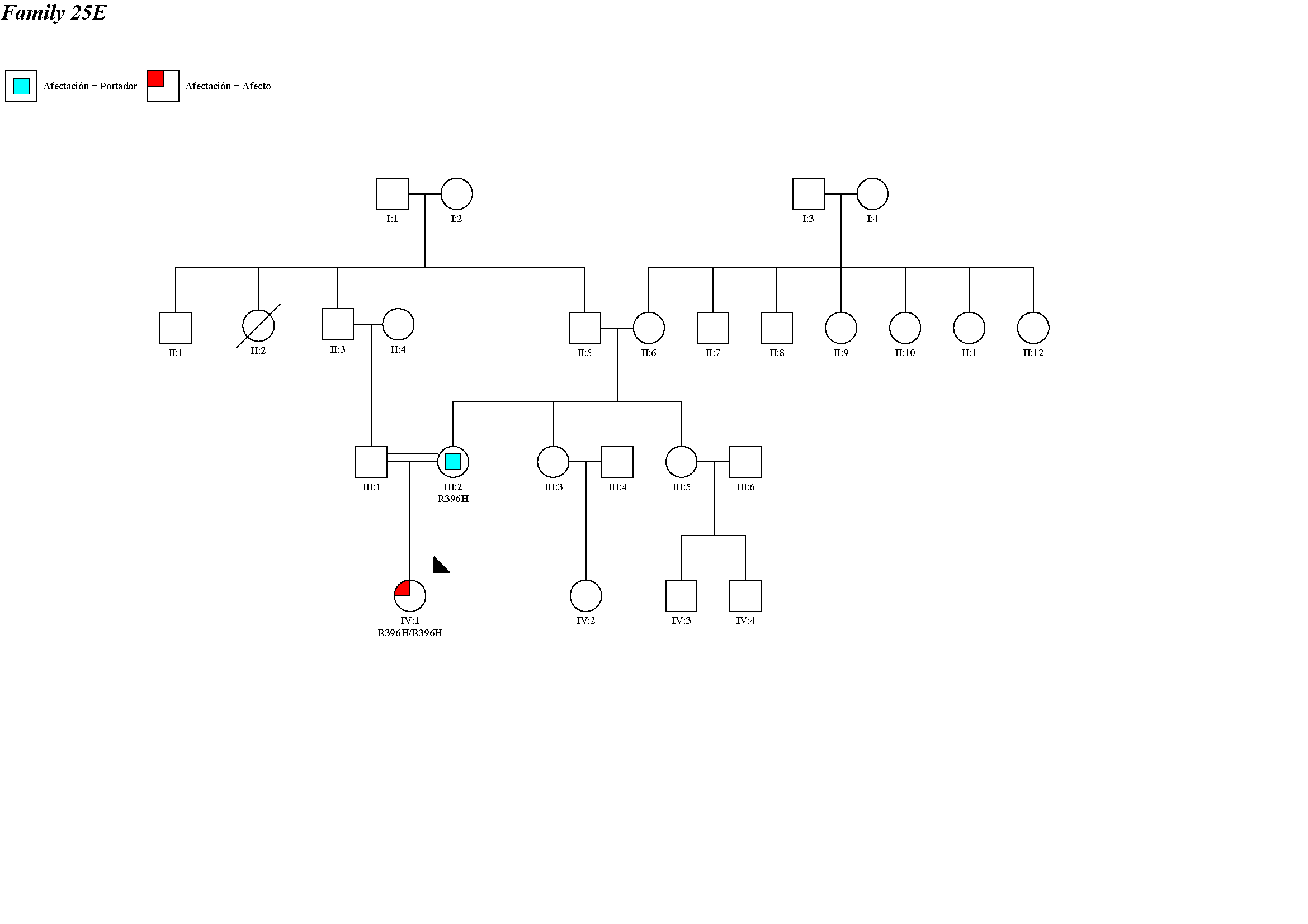
Figure S6. **Pedigree of family 25E.** Affected subjects and carriers are denoted with red and blue squares, respectively. The proband is marked with an upper left arrow.

Figure S7. **Pedigree of family 27E.** Affected subjects and carriers are denoted with red and blue squares, respectively. The proband is marked with an upper left arrow.


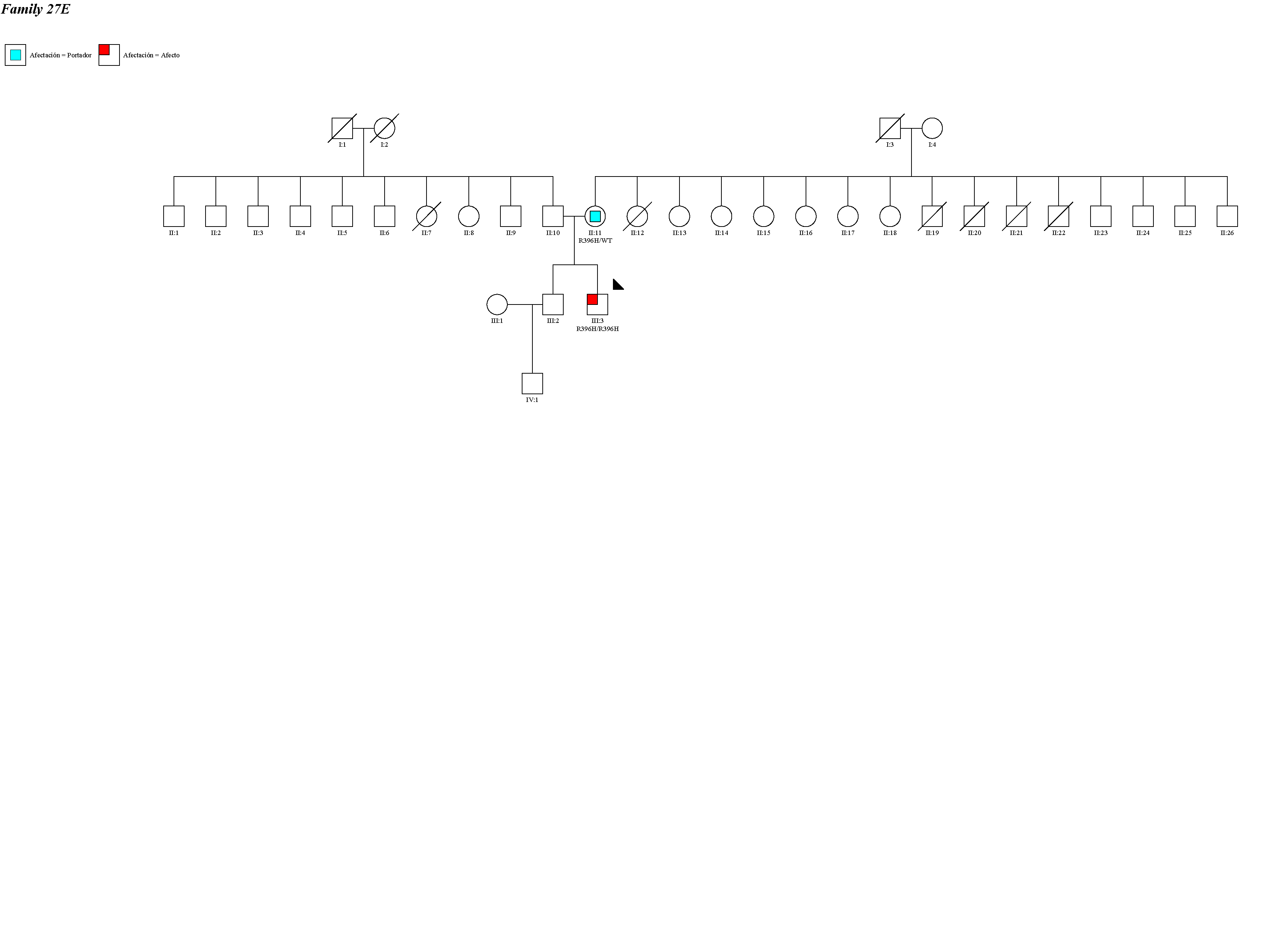


**Table S1.** TMRCA and mutation age estimations. Detailed information of each marker including: distance to the mutation in Mb, estimated recombination fraction, founder allele in cases and controls.

| **MICROSATELLITES** | | | | | | **TMRCA ESTIMATIONS** | | | | | | | | **MUTATION AGE ESTIMATION** |
| --- | --- | --- | --- | --- | --- | --- | --- | --- | --- | --- | --- | --- | --- | --- |
| ***Markers*** | ***Distance to mutation*** | ***Haldane (θ)*** | ***Founder allele*** | ***PD*** | ***PN*** | ***Labuda correction*** | ***Bergman*** | ***Bergman corrected*** | ***Labuda*** | ***Labuda corrected*** | ***Risch*** | ***Lander*** | ***Gamma method*** | ***DMLE*** |
|  |  |  |  |  |  |  |  |  |  |  |  |  |  |  |
| **D14S72** | 3,36 | 0,069 | 1 | 0,3 | 0,22 | 5 | 32 | 39 | 17 | 22 | 10176 | 8 | * | * |
| **D14S1043** | 3,35 | 0,069 | 5 | 0,6 | 0,83 | 8 | * | * | 13 | 21 | 5 | 17 | * | * |
| **D14S742** | 2,52 | 0,053 | 3 | 0,85 | 0,4 | 10 | 5 | 14 | 5 | 15 | 57 | 43 | * | * |
| **D14S581** | 0,43 | 0,009 | 3 | 0,45 | 0,29 | 20 | 155 | 173 | 150 | 170 | * | 81 | * | * |
| **D14S64** | 0,17 | 0,004 | 8 | 0,5 | 0,03 | 26 | 196 | 218 | 331 | 357 | * | 225 | * | * |
| **D14S264** | 0,55 | 0,005 | 8 | 1 | 0,27 | 19 | * | * | * | * | * | * | * | * |
| **D14S1032** | 1,48 | 0,032 | 8 | 0,75 | 0,12 | 13 | 10 | 22 | 16 | 29 | 16 | 56 | * | * |
| **D14S275** | 1,97 | 0,042 | 3 | 0,75 | 0,31 | 11 | 10 | 21 | 12 | 23 | 20 | 38 | * | * |
| **D14S1042** | 4,53 | 0,091 | 4 | 0,9 | 0,35 | 6 | 2 | 8 | 2 | 8 | * | 30 | * | * |
| **D14S1060** | 8,69 | 0,16 | 5 | 0,8 | 0,57 | 3 | 4 | 7 | 2 | 5 | 8 | 13 | * | * |
|  |  |  |  |  |  | ***Generations*** | *10 (5-10)* | *22 (11-22)* | *13 (5-13)* | *22 (15-22)* | *18 (8-18)* | *38 (17-38)* | *27(20-37)* | *41(32-58)* |

TMRCA calculations in generations for each marker and Labuda´s correction also in generations.

The botton row shows the median of the TMRCA age estimation for each estimator and the interquartile range (IQR)

Gamma method and DMLE results are given in number of generations with a confidence interval of 95%

θ: recombination fraction according to Haldane mapping function

PD: frequency of the founder allele in the normal population

PN: frequency of the founder allele in the disease population

| **Table S2**. Markers used for the ancestral study. | | | |  |  |
| --- | --- | --- | --- | --- | --- |
|  |  |  |  |  |  |
| **Chromosome** | **Start** | **End** | **Nº of markers** | **Nº of markers after LD filtering** | **Genes** |
| chr1 | 35225291 | 35225439 | 8 | 3 | GJB4 |
| chr1 | 35226483 | 35229375 | 156 | 48 | GJB4 |
| chr1 | 35246739 | 35247429 | 13 | 4 | GJB3 |
| chr1 | 35247828 | 35248093 | 10 | 4 | GJB3 |
| chr1 | 35250288 | 35252020 | 74 | 25 | GJB3 |
| chr1 | 152274600 | 152287273 | 1068 | 235 | FLG |
| chr1 | 152287744 | 152288003 | 6 | 0 | FLG |
| chr1 | 152297614 | 152297729 | 2 | 1 | FLG |
| chr1 | 153232125 | 153232259 | 3 | 0 | LOR |
| chr1 | 153233351 | 153234650 | 30 | 14 | LOR |
| chr1 | 155204188 | 155205685 | 41 | 14 | GBA |
| chr1 | 155205922 | 155206310 | 15 | 8 | GBA |
| chr1 | 155207081 | 155207419 | 5 | 0 | GBA |
| chr1 | 155207672 | 155208147 | 14 | 2 | GBA |
| chr1 | 155208257 | 155208732 | 10 | 4 | GBA |
| chr1 | 155209126 | 155209603 | 15 | 4 | GBA |
| chr1 | 155209626 | 155210140 | 16 | 4 | GBA |
| chr1 | 155210370 | 155210558 | 5 | 0 | GBA |
| chr1 | 155210826 | 155211119 | 9 | 3 | GBA |
| chr1 | 155213835 | 155214074 | 8 | 2 | GBA |
| chr1 | 155214246 | 155214703 | 18 | 7 | GBA |
| chr2 | 128014815 | 128015353 | 17 | 5 | ERCC3 |
| chr2 | 128016821 | 128020508 | 104 | 36 | ERCC3 |
| chr2 | 128028861 | 128029079 | 1 | 0 | ERCC3 |
| chr2 | 128030390 | 128030587 | 4 | 1 | ERCC3 |
| chr2 | 128036698 | 128037003 | 5 | 1 | ERCC3 |
| chr2 | 128037972 | 128038257 | 11 | 2 | ERCC3 |
| chr2 | 128044228 | 128044643 | 10 | 2 | ERCC3 |
| chr2 | 128046185 | 128046490 | 11 | 3 | ERCC3 |
| chr2 | 128046862 | 128048215 | 35 | 7 | ERCC3 |
| chr2 | 128049960 | 128050472 | 14 | 6 | ERCC3 |
| chr2 | 128051038 | 128051802 | 36 | 10 | ERCC3 |
| chr2 | 159813999 | 159963000 | 4049 | 578 | INTERGENIC |
| chr2 | 215796215 | 215797515 | 35 | 7 | ABCA12 |
| chr2 | 215798751 | 215798989 | 6 | 2 | ABCA12 |
| chr2 | 215802183 | 215802389 | 7 | 1 | ABCA12 |
| chr2 | 215807598 | 215807791 | 5 | 2 | ABCA12 |
| chr2 | 215809674 | 215809878 | 5 | 1 | ABCA12 |
| chr2 | 215812095 | 215812330 | 1 | 1 | ABCA12 |
| chr2 | 215813269 | 215813511 | 5 | 1 | ABCA12 |
| chr2 | 215813713 | 215813923 | 7 | 3 | ABCA12 |
| chr2 | 215815552 | 215815857 | 8 | 4 | ABCA12 |
| chr2 | 215818527 | 215818881 | 10 | 3 | ABCA12 |
| chr2 | 215819875 | 215820135 | 15 | 3 | ABCA12 |
| chr2 | 215821336 | 215821552 | 5 | 1 | ABCA12 |
| chr2 | 215822950 | 215823228 | 7 | 2 | ABCA12 |
| chr2 | 215823697 | 215823852 | 7 | 1 | ABCA12 |
| chr2 | 215831521 | 215831727 | 7 | 1 | ABCA12 |
| chr2 | 215833393 | 215833581 | 5 | 1 | ABCA12 |
| chr2 | 215834946 | 215835174 | 12 | 2 | ABCA12 |
| chr2 | 215838622 | 215838816 | 3 | 1 | ABCA12 |
| chr2 | 215839451 | 215839638 | 5 | 2 | ABCA12 |
| chr2 | 215840458 | 215840811 | 12 | 0 | ABCA12 |
| chr2 | 215842989 | 215843240 | 4 | 0 | ABCA12 |
| chr2 | 215843477 | 215843814 | 15 | 2 | ABCA12 |
| chr2 | 215845156 | 215845417 | 11 | 1 | ABCA12 |
| chr2 | 215846860 | 215847157 | 6 | 2 | ABCA12 |
| chr2 | 215848320 | 215848639 | 8 | 5 | ABCA12 |
| chr2 | 215851215 | 215851502 | 8 | 3 | ABCA12 |
| chr2 | 215852320 | 215852567 | 4 | 0 | ABCA12 |
| chr2 | 215854002 | 215854395 | 6 | 3 | ABCA12 |
| chr2 | 215855375 | 215855805 | 9 | 2 | ABCA12 |
| chr2 | 215862368 | 215862583 | 10 | 3 | ABCA12 |
| chr2 | 215865378 | 215865794 | 11 | 1 | ABCA12 |
| chr2 | 215866231 | 215866511 | 6 | 1 | ABCA12 |
| chr2 | 215868882 | 215869073 | 6 | 1 | ABCA12 |
| chr2 | 215872400 | 215872620 | 3 | 0 | ABCA12 |
| chr2 | 215875004 | 215875244 | 2 | 1 | ABCA12 |
| chr2 | 215876112 | 215876423 | 10 | 5 | ABCA12 |
| chr2 | 215876644 | 215876909 | 11 | 2 | ABCA12 |
| chr2 | 215880163 | 215880437 | 9 | 2 | ABCA12 |
| chr2 | 215882681 | 215882906 | 5 | 2 | ABCA12 |
| chr2 | 215884009 | 215884570 | 17 | 3 | ABCA12 |
| chr2 | 215890346 | 215890553 | 5 | 1 | ABCA12 |
| chr2 | 215891493 | 215891712 | 6 | 1 | ABCA12 |
| chr2 | 215896494 | 215896860 | 6 | 2 | ABCA12 |
| chr2 | 215901626 | 215901839 | 8 | 3 | ABCA12 |
| chr2 | 215910510 | 215910789 | 10 | 1 | ABCA12 |
| chr2 | 215914299 | 215914585 | 8 | 1 | ABCA12 |
| chr2 | 215917160 | 215917358 | 7 | 0 | ABCA12 |
| chr2 | 215919246 | 215919438 | 3 | 0 | ABCA12 |
| chr2 | 215928738 | 215928992 | 6 | 2 | ABCA12 |
| chr2 | 215940164 | 215940390 | 5 | 3 | ABCA12 |
| chr2 | 215976269 | 215976463 | 6 | 0 | ABCA12 |
| chr2 | 216002812 | 216003201 | 7 | 2 | ABCA12 |
| chr3 | 3742447 | 3744195 | 75 | 22 | INTERGENIC |
| chr3 | 3819358 | 3819537 | 7 | 2 | INTERGENIC |
| chr3 | 3820874 | 3821056 | 3 | 0 | INTERGENIC |
| chr3 | 3822985 | 3824435 | 65 | 26 | INTERGENIC |
| chr3 | 3830547 | 3830808 | 10 | 5 | INTERGENIC |
| chr3 | 3836872 | 3837162 | 11 | 4 | INTERGENIC |
| chr3 | 3839534 | 3839948 | 12 | 3 | INTERGENIC |
| chr3 | 3841345 | 3841630 | 5 | 3 | LRRN1 |
| chr3 | 3842334 | 3842609 | 7 | 4 | LRRN1 |
| chr3 | 3864367 | 3864631 | 11 | 1 | LRRN1 |
| chr3 | 3868875 | 3869243 | 9 | 2 | LRRN1 |
| chr3 | 4046822 | 4046930 | 3 | 1 | INTERGENIC |
| chr3 | 4110202 | 4110479 | 9 | 5 | INTERGENIC |
| chr3 | 4270943 | 4271082 | 7 | 3 | INTERGENIC |
| chr3 | 4402778 | 4403988 | 62 | 28 | SUMF1 |
| chr3 | 4417963 | 4418123 | 6 | 3 | SUMF1 |
| chr3 | 4452498 | 4452712 | 7 | 1 | SUMF1 |
| chr3 | 4458761 | 4458976 | 2 | 1 | SUMF1 |
| chr3 | 4459643 | 4459866 | 10 | 2 | SUMF1 |
| chr3 | 4461697 | 4461880 | 10 | 3 | SUMF1 |
| chr3 | 4490899 | 4491074 | 6 | 2 | SUMF1 |
| chr3 | 4494509 | 4494783 | 8 | 1 | SUMF1 |
| chr3 | 4508609 | 4509016 | 19 | 8 | SUMF1 |
| chr3 | 43731554 | 43731744 | 3 | 1 | ABHD5 |
| chr3 | 43731822 | 43732252 | 18 | 3 | ABHD5 |
| chr3 | 43732311 | 43732581 | 21 | 9 | ABHD5 |
| chr3 | 43732698 | 43733051 | 8 | 4 | ABHD5 |
| chr3 | 43740717 | 43741287 | 13 | 4 | ABHD5 |
| chr3 | 43743353 | 43743550 | 4 | 1 | ABHD5 |
| chr3 | 43743656 | 43744129 | 16 | 2 | ABHD5 |
| chr3 | 43753148 | 43753406 | 8 | 1 | ABHD5 |
| chr3 | 43756388 | 43756600 | 4 | 1 | ABHD5 |
| chr3 | 43759109 | 43759399 | 5 | 0 | ABHD5 |
| chr3 | 43759884 | 43764267 | 115 | 21 | ABHD5 |
| chr3 | 43775491 | 43775913 | 19 | 2 | ABHD5 |
| chr3 | 190023439 | 190026278 | 85 | 28 | CLDN1 |
| chr3 | 190027907 | 190028092 | 5 | 1 | CLDN1 |
| chr3 | 190030610 | 190031057 | 21 | 4 | CLDN1 |
| chr3 | 190039722 | 190040314 | 22 | 10 | CLDN1 |
| chr5 | 147405195 | 147405399 | 6 | 2 | SPINK5 |
| chr5 | 147443375 | 147443712 | 14 | 4 | SPINK5 |
| chr5 | 147444859 | 147445088 | 9 | 1 | SPINK5 |
| chr5 | 147449835 | 147450063 | 6 | 2 | SPINK5 |
| chr5 | 147451660 | 147451833 | 4 | 1 | SPINK5 |
| chr5 | 147465917 | 147466145 | 7 | 0 | SPINK5 |
| chr5 | 147468054 | 147468218 | 3 | 0 | SPINK5 |
| chr5 | 147468531 | 147468706 | 3 | 1 | SPINK5 |
| chr5 | 147468729 | 147468885 | 2 | 0 | SPINK5 |
| chr5 | 147469006 | 147469234 | 8 | 4 | SPINK5 |
| chr5 | 147470165 | 147470313 | 4 | 0 | SPINK5 |
| chr5 | 147470440 | 147470658 | 6 | 2 | SPINK5 |
| chr5 | 147470677 | 147470841 | 5 | 1 | SPINK5 |
| chr5 | 147473866 | 147474094 | 10 | 5 | SPINK5 |
| chr5 | 147475330 | 147475518 | 5 | 0 | SPINK5 |
| chr5 | 147477379 | 147477616 | 6 | 2 | SPINK5 |
| chr5 | 147478746 | 147478928 | 5 | 0 | SPINK5 |
| chr5 | 147479966 | 147480194 | 10 | 2 | SPINK5 |
| chr5 | 147480867 | 147481049 | 9 | 1 | SPINK5 |
| chr5 | 147481293 | 147481521 | 11 | 4 | SPINK5 |
| chr5 | 147484464 | 147484613 | 7 | 4 | SPINK5 |
| chr5 | 147486549 | 147486777 | 12 | 3 | SPINK5 |
| chr5 | 147488265 | 147488450 | 3 | 1 | SPINK5 |
| chr5 | 147491280 | 147491508 | 8 | 3 | SPINK5 |
| chr5 | 147492380 | 147492547 | 6 | 1 | SPINK5 |
| chr5 | 147493874 | 147494102 | 8 | 0 | SPINK5 |
| chr5 | 147495882 | 147496079 | 8 | 3 | SPINK5 |
| chr5 | 147497949 | 147498177 | 6 | 2 | SPINK5 |
| chr5 | 147498498 | 147498671 | 8 | 4 | SPINK5 |
| chr5 | 147499521 | 147499749 | 9 | 3 | SPINK5 |
| chr5 | 147499807 | 147500004 | 6 | 0 | SPINK5 |
| chr5 | 147503345 | 147503573 | 6 | 2 | SPINK5 |
| chr5 | 147504277 | 147504553 | 11 | 5 | SPINK5 |
| chr5 | 147505036 | 147505226 | 7 | 1 | SPINK5 |
| chr5 | 147505235 | 147505463 | 7 | 2 | SPINK5 |
| chr5 | 147506495 | 147506692 | 5 | 1 | SPINK5 |
| chr5 | 147510771 | 147511002 | 7 | 0 | SPINK5 |
| chr5 | 147513309 | 147513500 | 6 | 3 | SPINK5 |
| chr5 | 147516495 | 147516975 | 14 | 4 | SPINK5 |
| chr5 | 156886976 | 156887637 | 23 | 9 | NIPAL4 |
| chr5 | 156890051 | 156890391 | 15 | 4 | NIPAL4 |
| chr5 | 156894006 | 156894163 | 5 | 3 | NIPAL4 |
| chr5 | 156894214 | 156895026 | 22 | 9 | NIPAL4 |
| chr5 | 156895679 | 156895870 | 10 | 3 | NIPAL4 |
| chr5 | 156898614 | 156898875 | 8 | 1 | NIPAL4 |
| chr5 | 156899289 | 156901780 | 65 | 17 | NIPAL4 |
| chr6 | 36210894 | 36211088 | 4 | 2 | PNPLA1 |
| chr6 | 36238186 | 36238491 | 13 | 1 | PNPLA1 |
| chr6 | 36259046 | 36259382 | 19 | 10 | PNPLA1 |
| chr6 | 36260787 | 36260953 | 11 | 3 | PNPLA1 |
| chr6 | 36261889 | 36262226 | 11 | 3 | PNPLA1 |
| chr6 | 36263090 | 36263251 | 6 | 4 | PNPLA1 |
| chr6 | 36269587 | 36270296 | 33 | 8 | PNPLA1 |
| chr6 | 36274018 | 36274203 | 7 | 3 | PNPLA1 |
| chr6 | 36275313 | 36276422 | 37 | 19 | PNPLA1 |
| chr6 | 137143651 | 137143983 | 10 | 6 | PEX7 |
| chr6 | 137146301 | 137146459 | 2 | 1 | PEX7 |
| chr6 | 137147406 | 137147657 | 8 | 2 | PEX7 |
| chr6 | 137151094 | 137151448 | 6 | 4 | PEX7 |
| chr6 | 137166702 | 137166880 | 3 | 2 | PEX7 |
| chr6 | 137167160 | 137167369 | 4 | 1 | PEX7 |
| chr6 | 137187714 | 137187921 | 4 | 1 | PEX7 |
| chr6 | 137190977 | 137191191 | 5 | 2 | PEX7 |
| chr6 | 137191819 | 137192027 | 3 | 1 | PEX7 |
| chr6 | 137193285 | 137193441 | 2 | 2 | PEX7 |
| chr6 | 137219229 | 137219429 | 5 | 2 | PEX7 |
| chr6 | 137234545 | 137235122 | 17 | 6 | PEX7 |
| chr6 | 158589328 | 158589477 | 15 | 3 | GTF2H5 |
| chr6 | 158591451 | 158591620 | 5 | 1 | GTF2H5 |
| chr6 | 158612958 | 158620426 | 228 | 63 | GTF2H5 |
| chr7 | 40172291 | 40172908 | 13 | 4 | MPLKIP |
| chr7 | 40173777 | 40174308 | 15 | 4 | MPLKIP |
| chr7 | 100797627 | 100797983 | 21 | 9 | AP1S1 |
| chr7 | 100799824 | 100800103 | 6 | 3 | AP1S1 |
| chr7 | 100800607 | 100800816 | 7 | 2 | AP1S1 |
| chr7 | 100802289 | 100802527 | 9 | 3 | AP1S1 |
| chr7 | 100803749 | 100804608 | 27 | 8 | AP1S1 |
| chr7 | 100804722 | 100804927 | 5 | 3 | AP1S1 |
| chr9 | 131102788 | 131103167 | 13 | 5 | SLC27A4 |
| chr9 | 131105355 | 131105622 | 8 | 1 | SLC27A4 |
| chr9 | 131107383 | 131107878 | 19 | 8 | SLC27A4 |
| chr9 | 131110773 | 131111032 | 6 | 3 | SLC27A4 |
| chr9 | 131112540 | 131112710 | 7 | 3 | SLC27A4 |
| chr9 | 131112712 | 131112904 | 6 | 1 | SLC27A4 |
| chr9 | 131114866 | 131115076 | 4 | 1 | SLC27A4 |
| chr9 | 131115253 | 131115563 | 9 | 7 | SLC27A4 |
| chr9 | 131115643 | 131115870 | 6 | 0 | SLC27A4 |
| chr9 | 131117281 | 131117519 | 3 | 1 | SLC27A4 |
| chr9 | 131117626 | 131118125 | 19 | 5 | SLC27A4 |
| chr9 | 131122562 | 131123799 | 36 | 14 | SLC27A4 |
| chr10 | 13319745 | 13320404 | 17 | 5 | PHYH |
| chr10 | 13322925 | 13323160 | 8 | 1 | PHYH |
| chr10 | 13325639 | 13325889 | 7 | 2 | PHYH |
| chr10 | 13330309 | 13330591 | 13 | 3 | PHYH |
| chr10 | 13333410 | 13333962 | 18 | 10 | PHYH |
| chr10 | 13336377 | 13337037 | 27 | 8 | PHYH |
| chr10 | 13337177 | 13337656 | 15 | 4 | PHYH |
| chr10 | 13340136 | 13340295 | 8 | 4 | PHYH |
| chr10 | 13341369 | 13341813 | 21 | 8 | PHYH |
| chr10 | 13341917 | 13342180 | 6 | 3 | PHYH |
| chr10 | 13343935 | 13344462 | 13 | 5 | PHYH |
| chr10 | 90519901 | 90520057 | 5 | 2 | LIPN |
| chr10 | 90521104 | 90521320 | 1 | 0 | LIPN |
| chr10 | 90521894 | 90522112 | 5 | 1 | LIPN |
| chr10 | 90524116 | 90524415 | 8 | 3 | LIPN |
| chr10 | 90525975 | 90526185 | 3 | 1 | LIPN |
| chr10 | 90528498 | 90528735 | 11 | 5 | LIPN |
| chr10 | 90530551 | 90530798 | 9 | 4 | LIPN |
| chr10 | 90534179 | 90534351 | 2 | 2 | LIPN |
| chr10 | 90534798 | 90534970 | 7 | 4 | LIPN |
| chr10 | 90537715 | 90538049 | 14 | 7 | LIPN |
| chr11 | 130029406 | 130030139 | 18 | 7 | ST14 |
| chr11 | 130057958 | 130058218 | 11 | 4 | ST14 |
| chr11 | 130058374 | 130058602 | 9 | 8 | ST14 |
| chr11 | 130058713 | 130058884 | 7 | 2 | ST14 |
| chr11 | 130059169 | 130059841 | 27 | 12 | ST14 |
| chr11 | 130059885 | 130060639 | 32 | 11 | ST14 |
| chr11 | 130063993 | 130064233 | 10 | 3 | ST14 |
| chr11 | 130064484 | 130064682 | 10 | 4 | ST14 |
| chr11 | 130066183 | 130066393 | 11 | 3 | ST14 |
| chr11 | 130066414 | 130066739 | 12 | 3 | ST14 |
| chr11 | 130067685 | 130067890 | 12 | 5 | ST14 |
| chr11 | 130068152 | 130068566 | 24 | 9 | ST14 |
| chr11 | 130068791 | 130069014 | 11 | 3 | ST14 |
| chr11 | 130069795 | 130070082 | 8 | 2 | ST14 |
| chr11 | 130078254 | 130078629 | 12 | 3 | ST14 |
| chr11 | 130079286 | 130080406 | 35 | 13 | ST14 |
| chr12 | 53038291 | 53039403 | 47 | 18 | KRT2 |
| chr12 | 53040473 | 53040794 | 13 | 3 | KRT2 |
| chr12 | 53041463 | 53041689 | 7 | 2 | KRT2 |
| chr12 | 53041906 | 53042171 | 12 | 4 | KRT2 |
| chr12 | 53042740 | 53042936 | 11 | 4 | KRT2 |
| chr12 | 53043647 | 53043808 | 6 | 2 | KRT2 |
| chr12 | 53044072 | 53044506 | 12 | 1 | KRT2 |
| chr12 | 53045291 | 53046009 | 32 | 13 | KRT2 |
| chr12 | 53068469 | 53069578 | 48 | 12 | KRT1 |
| chr12 | 53070008 | 53070329 | 14 | 4 | KRT1 |
| chr12 | 53070792 | 53071018 | 5 | 2 | KRT1 |
| chr12 | 53071049 | 53071351 | 9 | 2 | KRT1 |
| chr12 | 53071383 | 53071579 | 4 | 1 | KRT1 |
| chr12 | 53071896 | 53072057 | 5 | 0 | KRT1 |
| chr12 | 53072275 | 53072590 | 6 | 2 | KRT1 |
| chr12 | 53073491 | 53074241 | 32 | 13 | KRT1 |
| chr13 | 20761553 | 20763969 | 99 | 32 | GJB2 |
| chr13 | 20766871 | 20767164 | 6 | 5 | GJB2 |
| chr13 | 20796050 | 20797684 | 56 | 17 | GJB6 |
| chr13 | 20803668 | 20803938 | 8 | 3 | GJB6 |
| chr13 | 20804786 | 20805422 | 21 | 9 | GJB6 |
| chr13 | 20805470 | 20805694 | 4 | 2 | GJB6 |
| chr13 | 20806282 | 20806584 | 6 | 4 | GJB6 |
| chr13 | 29233090 | 29233374 | 17 | 5 | POMP |
| chr13 | 29233908 | 29234106 | 6 | 3 | POMP |
| chr13 | 29236496 | 29236694 | 5 | 1 | POMP |
| chr13 | 29238595 | 29238756 | 1 | 0 | POMP |
| chr13 | 29242559 | 29242761 | 6 | 1 | POMP |
| chr13 | 29246425 | 29246619 | 5 | 0 | POMP |
| chr13 | 29252121 | 29253143 | 26 | 4 | POMP |
| chr14 | 24718269 | 24718797 | 13 | 7 | TGM1 |
| chr14 | 24723278 | 24723544 | 11 | 3 | TGM1 |
| chr14 | 24723819 | 24724080 | 9 | 6 | TGM1 |
| chr14 | 24724127 | 24724509 | 13 | 3 | TGM1 |
| chr14 | 24724519 | 24724773 | 12 | 5 | TGM1 |
| chr14 | 24725144 | 24725333 | 4 | 0 | TGM1 |
| chr14 | 24727440 | 24727644 | 4 | 1 | TGM1 |
| chr14 | 24727690 | 24727929 | 9 | 4 | TGM1 |
| chr14 | 24728230 | 24728505 | 12 | 5 | TGM1 |
| chr14 | 24728859 | 24729067 | 10 | 3 | TGM1 |
| chr14 | 24729095 | 24729414 | 13 | 7 | TGM1 |
| chr14 | 24729605 | 24729954 | 18 | 1 | TGM1 |
| chr14 | 24730850 | 24731139 | 21 | 10 | TGM1 |
| chr14 | 24731189 | 24731610 | 28 | 12 | TGM1 |
| chr14 | 24732244 | 24732796 | 24 | 10 | TGM1 |
| chr14 | 24733089 | 24733688 | 15 | 6 | TGM1 |
| chr15 | 91541595 | 91542334 | 27 | 8 | VPS33B |
| chr15 | 91542510 | 91543073 | 20 | 4 | VPS33B |
| chr15 | 91543079 | 91543255 | 3 | 2 | VPS33B |
| chr15 | 91543669 | 91544285 | 16 | 8 | VPS33B |
| chr15 | 91544568 | 91544742 | 1 | 1 | VPS33B |
| chr15 | 91545229 | 91545462 | 8 | 2 | VPS33B |
| chr15 | 91546264 | 91546411 | 3 | 1 | VPS33B |
| chr15 | 91548056 | 91548211 | 5 | 1 | VPS33B |
| chr15 | 91548234 | 91548399 | 6 | 0 | VPS33B |
| chr15 | 91548559 | 91548734 | 6 | 4 | VPS33B |
| chr15 | 91548873 | 91549064 | 7 | 2 | VPS33B |
| chr15 | 91549152 | 91549339 | 7 | 2 | VPS33B |
| chr15 | 91549551 | 91550326 | 28 | 10 | VPS33B |
| chr15 | 91550648 | 91550853 | 6 | 1 | VPS33B |
| chr15 | 91551049 | 91551244 | 6 | 0 | VPS33B |
| chr15 | 91552980 | 91553126 | 3 | 0 | VPS33B |
| chr15 | 91556951 | 91557151 | 9 | 3 | VPS33B |
| chr15 | 91557563 | 91557713 | 5 | 3 | VPS33B |
| chr15 | 91560142 | 91560344 | 7 | 3 | VPS33B |
| chr15 | 91560984 | 91561165 | 5 | 0 | VPS33B |
| chr15 | 91561197 | 91561316 | 2 | 1 | VPS33B |
| chr15 | 91565333 | 91565883 | 20 | 7 | VPS33B |
| chr15 | 100940549 | 100943120 | 93 | 29 | CERS3 |
| chr15 | 100996047 | 100996301 | 11 | 7 | CERS3 |
| chr15 | 101009532 | 101009739 | 7 | 2 | CERS3 |
| chr15 | 101013078 | 101013307 | 11 | 5 | CERS3 |
| chr15 | 101015991 | 101016433 | 12 | 3 | CERS3 |
| chr15 | 101019582 | 101019751 | 4 | 1 | CERS3 |
| chr15 | 101020143 | 101020301 | 0 | 0 | CERS3 |
| chr15 | 101024704 | 101024923 | 10 | 2 | CERS3 |
| chr15 | 101030926 | 101031186 | 8 | 5 | CERS3 |
| chr15 | 101040402 | 101040759 | 10 | 3 | CERS3 |
| chr15 | 101041831 | 101042105 | 16 | 4 | CERS3 |
| chr15 | 101043864 | 101044103 | 10 | 4 | CERS3 |
| chr15 | 101061821 | 101062011 | 2 | 1 | CERS3 |
| chr15 | 101068967 | 101069520 | 15 | 10 | CERS3 |
| chr15 | 101084278 | 101084668 | 15 | 8 | CERS3 |
| chr15 | 101084805 | 101084975 | 7 | 2 | CERS3 |
| chr15 | 101085057 | 101085250 | 11 | 3 | CERS3 |
| chr17 | 7975903 | 7976318 | 8 | 5 | ALOX12B |
| chr17 | 7976415 | 7976686 | 5 | 1 | ALOX12B |
| chr17 | 7976924 | 7977125 | 6 | 3 | ALOX12B |
| chr17 | 7978862 | 7979084 | 7 | 5 | ALOX12B |
| chr17 | 7979442 | 7979712 | 11 | 3 | ALOX12B |
| chr17 | 7979924 | 7980111 | 1 | 0 | ALOX12B |
| chr17 | 7980257 | 7980561 | 13 | 6 | ALOX12B |
| chr17 | 7982663 | 7982907 | 6 | 3 | ALOX12B |
| chr17 | 7983036 | 7983463 | 10 | 5 | ALOX12B |
| chr17 | 7983502 | 7983706 | 9 | 1 | ALOX12B |
| chr17 | 7983925 | 7984148 | 5 | 2 | ALOX12B |
| chr17 | 7984151 | 7984344 | 12 | 3 | ALOX12B |
| chr17 | 7984373 | 7984555 | 5 | 2 | ALOX12B |
| chr17 | 7989283 | 7989588 | 13 | 6 | ALOX12B |
| chr17 | 7990563 | 7991072 | 20 | 8 | ALOX12B |
| chr17 | 7999167 | 8000174 | 40 | 14 | ALOXE3 |
| chr17 | 8006590 | 8006883 | 10 | 2 | ALOXE3 |
| chr17 | 8007382 | 8007583 | 4 | 2 | ALOXE3 |
| chr17 | 8011735 | 8011957 | 7 | 0 | ALOXE3 |
| chr17 | 8012441 | 8012711 | 9 | 4 | ALOXE3 |
| chr17 | 8013183 | 8013896 | 23 | 4 | ALOXE3 |
| chr17 | 8014626 | 8014899 | 8 | 1 | ALOXE3 |
| chr17 | 8015360 | 8015564 | 4 | 1 | ALOXE3 |
| chr17 | 8017751 | 8017977 | 15 | 3 | ALOXE3 |
| chr17 | 8018205 | 8018425 | 7 | 3 | ALOXE3 |
| chr17 | 8018874 | 8019056 | 3 | 3 | ALOXE3 |
| chr17 | 8020043 | 8020348 | 13 | 3 | ALOXE3 |
| chr17 | 8021111 | 8021910 | 17 | 9 | ALOXE3 |
| chr17 | 8021963 | 8022415 | 19 | 7 | ALOXE3 |
| chr17 | 19551398 | 19551628 | 5 | 3 | ALDH3A2 |
| chr17 | 19551788 | 19552487 | 28 | 10 | ALDH3A2 |
| chr17 | 19554809 | 19555141 | 8 | 0 | ALDH3A2 |
| chr17 | 19555809 | 19556060 | 8 | 1 | ALDH3A2 |
| chr17 | 19558543 | 19558750 | 3 | 1 | ALDH3A2 |
| chr17 | 19559407 | 19559937 | 17 | 4 | ALDH3A2 |
| chr17 | 19560983 | 19561225 | 4 | 1 | ALDH3A2 |
| chr17 | 19562835 | 19563062 | 8 | 6 | ALDH3A2 |
| chr17 | 19564389 | 19564805 | 10 | 2 | ALDH3A2 |
| chr17 | 19566595 | 19566865 | 5 | 2 | ALDH3A2 |
| chr17 | 19568210 | 19568410 | 8 | 2 | ALDH3A2 |
| chr17 | 19574983 | 19575319 | 6 | 1 | ALDH3A2 |
| chr17 | 19575614 | 19575794 | 3 | 3 | ALDH3A2 |
| chr17 | 19576413 | 19576638 | 6 | 0 | ALDH3A2 |
| chr17 | 19577346 | 19580961 | 81 | 20 | ALDH3A2 |
| chr17 | 38974318 | 38974784 | 14 | 6 | KRT10 |
| chr17 | 38974988 | 38975463 | 15 | 6 | KRT10 |
| chr17 | 38975718 | 38976093 | 6 | 2 | KRT10 |
| chr17 | 38976250 | 38976969 | 15 | 3 | KRT10 |
| chr17 | 38977236 | 38977419 | 4 | 0 | KRT10 |
| chr17 | 38978160 | 38978913 | 28 | 9 | KRT10 |
| chr19 | 15619285 | 15619444 | 4 | 3 | CYP4F22 |
| chr19 | 15634453 | 15634660 | 4 | 2 | CYP4F22 |
| chr19 | 15636093 | 15636419 | 19 | 9 | CYP4F22 |
| chr19 | 15640469 | 15640714 | 9 | 2 | CYP4F22 |
| chr19 | 15648121 | 15648275 | 7 | 3 | CYP4F22 |
| chr19 | 15648295 | 15648523 | 8 | 4 | CYP4F22 |
| chr19 | 15648632 | 15648854 | 11 | 4 | CYP4F22 |
| chr19 | 15651210 | 15651578 | 24 | 8 | CYP4F22 |
| chr19 | 15654731 | 15654898 | 5 | 1 | CYP4F22 |
| chr19 | 15654910 | 15655140 | 2 | 0 | CYP4F22 |
| chr19 | 15658868 | 15659102 | 10 | 3 | CYP4F22 |
| chr19 | 15659898 | 15660063 | 5 | 2 | CYP4F22 |
| chr19 | 15661434 | 15661617 | 2 | 2 | CYP4F22 |
| chr19 | 15662054 | 15663178 | 33 | 15 | CYP4F22 |
| chr19 | 45854195 | 45855029 | 64 | 21 | ERCC2 |
| chr19 | 45855416 | 45855660 | 19 | 5 | ERCC2 |
| chr19 | 45855713 | 45856124 | 24 | 4 | ERCC2 |
| chr19 | 45856290 | 45856642 | 20 | 6 | ERCC2 |
| chr19 | 45857937 | 45858159 | 10 | 2 | ERCC2 |
| chr19 | 45858872 | 45859036 | 5 | 1 | ERCC2 |
| chr19 | 45860477 | 45860679 | 8 | 1 | ERCC2 |
| chr19 | 45860681 | 45861007 | 11 | 2 | ERCC2 |
| chr19 | 45861972 | 45862220 | 16 | 5 | ERCC2 |
| chr19 | 45864731 | 45864950 | 9 | 3 | ERCC2 |
| chr19 | 45866950 | 45867427 | 19 | 4 | ERCC2 |
| chr19 | 45867442 | 45867855 | 19 | 7 | ERCC2 |
| chr19 | 45868045 | 45868466 | 14 | 5 | ERCC2 |
| chr19 | 45871837 | 45872051 | 9 | 1 | ERCC2 |
| chr19 | 45872137 | 45872455 | 8 | 2 | ERCC2 |
| chr19 | 45873340 | 45873926 | 20 | 5 | ERCC2 |
| chr22 | 21213220 | 21213685 | 26 | 7 | SNAP29 |
| chr22 | 21213720 | 21213978 | 6 | 2 | SNAP29 |
| chr22 | 21224574 | 21225379 | 21 | 7 | SNAP29 |
| chr22 | 21235286 | 21235472 | 5 | 2 | SNAP29 |
| chr22 | 21237708 | 21237907 | 3 | 0 | SNAP29 |
| chr22 | 21241916 | 21245552 | 132 | 38 | SNAP29 |
