# Supplementary material for: Biogeographical origin and timing of the founder ichthyosis TGM1 c.1187G > A mutation in an isolated Ecuadorian population
Source: Sci Rep. 2019 May 9;9:7175. doi: 10.1038/s41598-019-43133-6 (PMC6509209; doi:10.1038/s41598-019-43133-6)
Supplement: Supplementary file 2 — Table S1 [file 41598_2019_43133_MOESM2_ESM.pdf]

Table S1. TMRCA and mutation age estimations. Detailed information of each marker including: distance to the mutation in Mb, estimated recombination fraction, founder allele in cases and controls.

MICROSATELLITES						TMRCA ESTIMATIONS								MUTATION AGE ESTIMATION
Markers	*Distance to mutation*	*Haldane (θ)*	*Founder allele*	*PD*	*PN*	*Labuda correction*	*Bergman*	*Bergman corrected*	*Labuda*	*Labuda corrected*	*Risch*	*Lander*	*Gamma method*	*DMLE*
**D14S72**	3.36	0.069	1	0.3	0.2	5	32	39	17	22	10176	8	*	*
**D14S1043**	3.35	0.069	5	0.6	0.8	8	*	*	13	21	5	17	*	*
**D14S742**	2.52	0.053	3	0.9	0.4	10	5	14	5	15	57	43	*	*
**D14S581**	0.43	0.009	3	0.5	0.3	20	155	173	150	170	*	81	*	*
**D14S64**	0.17	0.004	8	0.5	0	26	196	218	331	357	*	225	*	*
**D14S264**	0.55	0.005	8	1	0.3	19	*	*	*	*	*	*	*	*
**D14S1032**	1.48	0.032	8	0.8	0.1	13	10	22	16	29	16	56	*	*
**D14S275**	1.97	0.042	3	0.8	0.3	11	10	21	12	23	20	38	*	*
**D14S1042**	4.53	0.091	4	0.9	0.4	6	2	8	2	8	*	30	*	*
**D14S1060**	8.69	0.16	5	0.8	0.6	3	4	7	2	5	8	13	*	*
						Generations	*10 (5-10)*	*22 (11-22)*	*13 (5-13)*	*22 (15-22)*	*18 (8-18)*	*38 (17-38)*	*27(20-37)*	*41(32-58)*

TMRCA calculations in generations for each marker and Labuda´s correction also in generations.
The botton row shows the median of the TMRCA age estimation for each estimator and the interquartile range (IQR)
Gamma method and DMLE results are given in number of generations with a confidence interval of 95%
θ: recombination fraction according to Haldane mapping function
PD: frequency of the founder allele in the normal population
PN: frequency of the founder allele in the disease population
