# Supplementary material for: Biogeographical origin and timing of the founder ichthyosis TGM1 c.1187G > A mutation in an isolated Ecuadorian population
Source: Sci Rep. 2019 May 9;9:7175. doi: 10.1038/s41598-019-43133-6 (PMC6509209; doi:10.1038/s41598-019-43133-6)
Supplement: Supplementary file 3 — Table S2 [file 41598_2019_43133_MOESM3_ESM.pdf]

**Table S2**. Markers used for the ancestral study.

| Chromosome | Start | End | Nº of markers | Nº of markers after LD filtering | Genes |
|---|---|---|---|---|---|
| chr1 | 35225291 | 35225439 | 8 | 3 | GJB4 |
| chr1 | 35226483 | 35229375 | 156 | 48 | GJB4 |
| chr1 | 35246739 | 35247429 | 13 | 4 | GJB3 |
| chr1 | 35247828 | 35248093 | 10 | 4 | GJB3 |
| chr1 | 35250288 | 35252020 | 74 | 25 | GJB3 |
| chr1 | 152274600 | 152287273 | 1068 | 235 | FLG |
| chr1 | 152287744 | 152288003 | 6 | 0 | FLG |
| chr1 | 152297614 | 152297729 | 2 | 1 | FLG |
| chr1 | 153232125 | 153232259 | 3 | 0 | LOR |
| chr1 | 153233351 | 153234650 | 30 | 14 | LOR |
| chr1 | 155204188 | 155205685 | 41 | 14 | GBA |
| chr1 | 155205922 | 155206310 | 15 | 8 | GBA |
| chr1 | 155207081 | 155207419 | 5 | 0 | GBA |
| chr1 | 155207672 | 155208147 | 14 | 2 | GBA |
| chr1 | 155208257 | 155208732 | 10 | 4 | GBA |
| chr1 | 155209126 | 155209603 | 15 | 4 | GBA |
| chr1 | 155209626 | 155210140 | 16 | 4 | GBA |
| chr1 | 155210370 | 155210558 | 5 | 0 | GBA |
| chr1 | 155210826 | 155211119 | 9 | 3 | GBA |
| chr1 | 155213835 | 155214074 | 8 | 2 | GBA |
| chr1 | 155214246 | 155214703 | 18 | 7 | GBA |
| chr2 | 128014815 | 128015353 | 17 | 5 | ERCC3 |
| chr2 | 128016821 | 128020508 | 104 | 36 | ERCC3 |
| chr2 | 128028861 | 128029079 | 1 | 0 | ERCC3 |
| chr2 | 128030390 | 128030587 | 4 | 1 | ERCC3 |
| chr2 | 128036698 | 128037003 | 5 | 1 | ERCC3 |
| chr2 | 128037972 | 128038257 | 11 | 2 | ERCC3 |
| chr2 | 128044228 | 128044643 | 10 | 2 | ERCC3 |
| chr2 | 128046185 | 128046490 | 11 | 3 | ERCC3 |
| chr2 | 128046862 | 128048215 | 35 | 7 | ERCC3 |
| chr2 | 128049960 | 128050472 | 14 | 6 | ERCC3 |
| chr2 | 128051038 | 128051802 | 36 | 10 | ERCC3 |
| chr2 | 159813999 | 159963000 | 4049 | 578 | INTERGENIC |
| chr2 | 215796215 | 215797515 | 35 | 7 | ABCA12 |
| chr2 | 215798751 | 215798989 | 6 | 2 | ABCA12 |
| chr2 | 215802183 | 215802389 | 7 | 1 | ABCA12 |
| chr2 | 215807598 | 215807791 | 5 | 2 | ABCA12 |
| chr2 | 215809674 | 215809878 | 5 | 1 | ABCA12 |
| chr2 | 215812095 | 215812330 | 1 | 1 | ABCA12 |
| chr2 | 215813269 | 215813511 | 5 | 1 | ABCA12 |
| chr2 | 215813713 | 215813923 | 7 | 3 | ABCA12 |
| chr2 | 215815552 | 215815857 | 8 | 4 | ABCA12 |
| chr2 | 215818527 | 215818881 | 10 | 3 | ABCA12 |
| chr2 | 215819875 | 215820135 | 15 | 3 | ABCA12 |
| chr2 | 215821336 | 215821552 | 5 | 1 | ABCA12 |
| chr2 | 215822950 | 215823228 | 7 | 2 | ABCA12 |
| chr2 | 215823697 | 215823852 | 7 | 1 | ABCA12 |
| chr2 | 215831521 | 215831727 | 7 | 1 | ABCA12 |
| chr2 | 215833393 | 215833581 | 5 | 1 | ABCA12 |
| chr2 | 215834946 | 215835174 | 12 | 2 | ABCA12 |

| | | | | | |
|---|---|---|---|---|---|
| chr2 | 215838622 | 215838816 | 3 | 1 | ABCA12 |
| chr2 | 215839451 | 215839638 | 5 | 2 | ABCA12 |
| chr2 | 215840458 | 215840811 | 12 | 0 | ABCA12 |
| chr2 | 215842989 | 215843240 | 4 | 0 | ABCA12 |
| chr2 | 215843477 | 215843814 | 15 | 2 | ABCA12 |
| chr2 | 215845156 | 215845417 | 11 | 1 | ABCA12 |
| chr2 | 215846860 | 215847157 | 6 | 2 | ABCA12 |
| chr2 | 215848320 | 215848639 | 8 | 5 | ABCA12 |
| chr2 | 215851215 | 215851502 | 8 | 3 | ABCA12 |
| chr2 | 215852320 | 215852567 | 4 | 0 | ABCA12 |
| chr2 | 215854002 | 215854395 | 6 | 3 | ABCA12 |
| chr2 | 215855375 | 215855805 | 9 | 2 | ABCA12 |
| chr2 | 215862368 | 215862583 | 10 | 3 | ABCA12 |
| chr2 | 215865378 | 215865794 | 11 | 1 | ABCA12 |
| chr2 | 215866231 | 215866511 | 6 | 1 | ABCA12 |
| chr2 | 215868882 | 215869073 | 6 | 1 | ABCA12 |
| chr2 | 215872400 | 215872620 | 3 | 0 | ABCA12 |
| chr2 | 215875004 | 215875244 | 2 | 1 | ABCA12 |
| chr2 | 215876112 | 215876423 | 10 | 5 | ABCA12 |
| chr2 | 215876644 | 215876909 | 11 | 2 | ABCA12 |
| chr2 | 215880163 | 215880437 | 9 | 2 | ABCA12 |
| chr2 | 215882681 | 215882906 | 5 | 2 | ABCA12 |
| chr2 | 215884009 | 215884570 | 17 | 3 | ABCA12 |
| chr2 | 215890346 | 215890553 | 5 | 1 | ABCA12 |
| chr2 | 215891493 | 215891712 | 6 | 1 | ABCA12 |
| chr2 | 215896494 | 215896860 | 6 | 2 | ABCA12 |
| chr2 | 215901626 | 215901839 | 8 | 3 | ABCA12 |
| chr2 | 215910510 | 215910789 | 10 | 1 | ABCA12 |
| chr2 | 215914299 | 215914585 | 8 | 1 | ABCA12 |
| chr2 | 215917160 | 215917358 | 7 | 0 | ABCA12 |
| chr2 | 215919246 | 215919438 | 3 | 0 | ABCA12 |
| chr2 | 215928738 | 215928992 | 6 | 2 | ABCA12 |
| chr2 | 215940164 | 215940390 | 5 | 3 | ABCA12 |
| chr2 | 215976269 | 215976463 | 6 | 0 | ABCA12 |
| chr2 | 216002812 | 216003201 | 7 | 2 | ABCA12 |
| chr3 | 3742447 | 3744195 | 75 | 22 | INTERGENIC |
| chr3 | 3819358 | 3819537 | 7 | 2 | INTERGENIC |
| chr3 | 3820874 | 3821056 | 3 | 0 | INTERGENIC |
| chr3 | 3822985 | 3824435 | 65 | 26 | INTERGENIC |
| chr3 | 3830547 | 3830808 | 10 | 5 | INTERGENIC |
| chr3 | 3836872 | 3837162 | 11 | 4 | INTERGENIC |
| chr3 | 3839534 | 3839948 | 12 | 3 | INTERGENIC |
| chr3 | 3841345 | 3841630 | 5 | 3 | LRRN1 |
| chr3 | 3842334 | 3842609 | 7 | 4 | LRRN1 |
| chr3 | 3864367 | 3864631 | 11 | 1 | LRRN1 |
| chr3 | 3868875 | 3869243 | 9 | 2 | LRRN1 |
| chr3 | 4046822 | 4046930 | 3 | 1 | INTERGENIC |
| chr3 | 4110202 | 4110479 | 9 | 5 | INTERGENIC |
| chr3 | 4270943 | 4271082 | 7 | 3 | INTERGENIC |
| chr3 | 4402778 | 4403988 | 62 | 28 | SUMF1 |
| chr3 | 4417963 | 4418123 | 6 | 3 | SUMF1 |
| chr3 | 4452498 | 4452712 | 7 | 1 | SUMF1 |
| chr3 | 4458761 | 4458976 | 2 | 1 | SUMF1 |

| | | | | | |
|---|---|---|---|---|---|
| chr3 | 4459643 | 4459866 | 10 | 2 | SUMF1 |
| chr3 | 4461697 | 4461880 | 10 | 3 | SUMF1 |
| chr3 | 4490899 | 4491074 | 6 | 2 | SUMF1 |
| chr3 | 4494509 | 4494783 | 8 | 1 | SUMF1 |
| chr3 | 4508609 | 4509016 | 19 | 8 | SUMF1 |
| chr3 | 43731554 | 43731744 | 3 | 1 | ABHD5 |
| chr3 | 43731822 | 43732252 | 18 | 3 | ABHD5 |
| chr3 | 43732311 | 43732581 | 21 | 9 | ABHD5 |
| chr3 | 43732698 | 43733051 | 8 | 4 | ABHD5 |
| chr3 | 43740717 | 43741287 | 13 | 4 | ABHD5 |
| chr3 | 43743353 | 43743550 | 4 | 1 | ABHD5 |
| chr3 | 43743656 | 43744129 | 16 | 2 | ABHD5 |
| chr3 | 43753148 | 43753406 | 8 | 1 | ABHD5 |
| chr3 | 43756388 | 43756600 | 4 | 1 | ABHD5 |
| chr3 | 43759109 | 43759399 | 5 | 0 | ABHD5 |
| chr3 | 43759884 | 43764267 | 115 | 21 | ABHD5 |
| chr3 | 43775491 | 43775913 | 19 | 2 | ABHD5 |
| chr3 | 190023439 | 190026278 | 85 | 28 | CLDN1 |
| chr3 | 190027907 | 190028092 | 5 | 1 | CLDN1 |
| chr3 | 190030610 | 190031057 | 21 | 4 | CLDN1 |
| chr3 | 190039722 | 190040314 | 22 | 10 | CLDN1 |
| chr5 | 147405195 | 147405399 | 6 | 2 | SPINK5 |
| chr5 | 147443375 | 147443712 | 14 | 4 | SPINK5 |
| chr5 | 147444859 | 147445088 | 9 | 1 | SPINK5 |
| chr5 | 147449835 | 147450063 | 6 | 2 | SPINK5 |
| chr5 | 147451660 | 147451833 | 4 | 1 | SPINK5 |
| chr5 | 147465917 | 147466145 | 7 | 0 | SPINK5 |
| chr5 | 147468054 | 147468218 | 3 | 0 | SPINK5 |
| chr5 | 147468531 | 147468706 | 3 | 1 | SPINK5 |
| chr5 | 147468729 | 147468885 | 2 | 0 | SPINK5 |
| chr5 | 147469006 | 147469234 | 8 | 4 | SPINK5 |
| chr5 | 147470165 | 147470313 | 4 | 0 | SPINK5 |
| chr5 | 147470440 | 147470658 | 6 | 2 | SPINK5 |
| chr5 | 147470677 | 147470841 | 5 | 1 | SPINK5 |
| chr5 | 147473866 | 147474094 | 10 | 5 | SPINK5 |
| chr5 | 147475330 | 147475518 | 5 | 0 | SPINK5 |
| chr5 | 147477379 | 147477616 | 6 | 2 | SPINK5 |
| chr5 | 147478746 | 147478928 | 5 | 0 | SPINK5 |
| chr5 | 147479966 | 147480194 | 10 | 2 | SPINK5 |
| chr5 | 147480867 | 147481049 | 9 | 1 | SPINK5 |
| chr5 | 147481293 | 147481521 | 11 | 4 | SPINK5 |
| chr5 | 147484464 | 147484613 | 7 | 4 | SPINK5 |
| chr5 | 147486549 | 147486777 | 12 | 3 | SPINK5 |
| chr5 | 147488265 | 147488450 | 3 | 1 | SPINK5 |
| chr5 | 147491280 | 147491508 | 8 | 3 | SPINK5 |
| chr5 | 147492380 | 147492547 | 6 | 1 | SPINK5 |
| chr5 | 147493874 | 147494102 | 8 | 0 | SPINK5 |
| chr5 | 147495882 | 147496079 | 8 | 3 | SPINK5 |
| chr5 | 147497949 | 147498177 | 6 | 2 | SPINK5 |
| chr5 | 147498498 | 147498671 | 8 | 4 | SPINK5 |
| chr5 | 147499521 | 147499749 | 9 | 3 | SPINK5 |
| chr5 | 147499807 | 147500004 | 6 | 0 | SPINK5 |
| chr5 | 147503345 | 147503573 | 6 | 2 | SPINK5 |

| | | | | | |
|---|---|---|---|---|---|
| chr5 | 147504277 | 147504553 | 11 | 5 | SPINK5 |
| chr5 | 147505036 | 147505226 | 7 | 1 | SPINK5 |
| chr5 | 147505235 | 147505463 | 7 | 2 | SPINK5 |
| chr5 | 147506495 | 147506692 | 5 | 1 | SPINK5 |
| chr5 | 147510771 | 147511002 | 7 | 0 | SPINK5 |
| chr5 | 147513309 | 147513500 | 6 | 3 | SPINK5 |
| chr5 | 147516495 | 147516975 | 14 | 4 | SPINK5 |
| chr5 | 156886976 | 156887637 | 23 | 9 | NIPAL4 |
| chr5 | 156890051 | 156890391 | 15 | 4 | NIPAL4 |
| chr5 | 156894006 | 156894163 | 5 | 3 | NIPAL4 |
| chr5 | 156894214 | 156895026 | 22 | 9 | NIPAL4 |
| chr5 | 156895679 | 156895870 | 10 | 3 | NIPAL4 |
| chr5 | 156898614 | 156898875 | 8 | 1 | NIPAL4 |
| chr5 | 156899289 | 156901780 | 65 | 17 | NIPAL4 |
| chr6 | 36210894 | 36211088 | 4 | 2 | PNPLA1 |
| chr6 | 36238186 | 36238491 | 13 | 1 | PNPLA1 |
| chr6 | 36259046 | 36259382 | 19 | 10 | PNPLA1 |
| chr6 | 36260787 | 36260953 | 11 | 3 | PNPLA1 |
| chr6 | 36261889 | 36262226 | 11 | 3 | PNPLA1 |
| chr6 | 36263090 | 36263251 | 6 | 4 | PNPLA1 |
| chr6 | 36269587 | 36270296 | 33 | 8 | PNPLA1 |
| chr6 | 36274018 | 36274203 | 7 | 3 | PNPLA1 |
| chr6 | 36275313 | 36276422 | 37 | 19 | PNPLA1 |
| chr6 | 137143651 | 137143983 | 10 | 6 | PEX7 |
| chr6 | 137146301 | 137146459 | 2 | 1 | PEX7 |
| chr6 | 137147406 | 137147657 | 8 | 2 | PEX7 |
| chr6 | 137151094 | 137151448 | 6 | 4 | PEX7 |
| chr6 | 137166702 | 137166880 | 3 | 2 | PEX7 |
| chr6 | 137167160 | 137167369 | 4 | 1 | PEX7 |
| chr6 | 137187714 | 137187921 | 4 | 1 | PEX7 |
| chr6 | 137190977 | 137191191 | 5 | 2 | PEX7 |
| chr6 | 137191819 | 137192027 | 3 | 1 | PEX7 |
| chr6 | 137193285 | 137193441 | 2 | 2 | PEX7 |
| chr6 | 137219229 | 137219429 | 5 | 2 | PEX7 |
| chr6 | 137234545 | 137235122 | 17 | 6 | PEX7 |
| chr6 | 158589328 | 158589477 | 15 | 3 | GTF2H5 |
| chr6 | 158591451 | 158591620 | 5 | 1 | GTF2H5 |
| chr6 | 158612958 | 158620426 | 228 | 63 | GTF2H5 |
| chr7 | 40172291 | 40172908 | 13 | 4 | MPLKIP |
| chr7 | 40173777 | 40174308 | 15 | 4 | MPLKIP |
| chr7 | 100797627 | 100797983 | 21 | 9 | AP1S1 |
| chr7 | 100799824 | 100800103 | 6 | 3 | AP1S1 |
| chr7 | 100800607 | 100800816 | 7 | 2 | AP1S1 |
| chr7 | 100802289 | 100802527 | 9 | 3 | AP1S1 |
| chr7 | 100803749 | 100804608 | 27 | 8 | AP1S1 |
| chr7 | 100804722 | 100804927 | 5 | 3 | AP1S1 |
| chr9 | 131102788 | 131103167 | 13 | 5 | SLC27A4 |
| chr9 | 131105355 | 131105622 | 8 | 1 | SLC27A4 |
| chr9 | 131107383 | 131107878 | 19 | 8 | SLC27A4 |
| chr9 | 131110773 | 131111032 | 6 | 3 | SLC27A4 |
| chr9 | 131112540 | 131112710 | 7 | 3 | SLC27A4 |
| chr9 | 131112712 | 131112904 | 6 | 1 | SLC27A4 |
| chr9 | 131114866 | 131115076 | 4 | 1 | SLC27A4 |

| | | | | | |
|---|---|---|---|---|---|
| chr9 | 131115253 | 131115563 | 9 | 7 | SLC27A4 |
| chr9 | 131115643 | 131115870 | 6 | 0 | SLC27A4 |
| chr9 | 131117281 | 131117519 | 3 | 1 | SLC27A4 |
| chr9 | 131117626 | 131118125 | 19 | 5 | SLC27A4 |
| chr9 | 131122562 | 131123799 | 36 | 14 | SLC27A4 |
| chr10 | 13319745 | 13320404 | 17 | 5 | PHYH |
| chr10 | 13322925 | 13323160 | 8 | 1 | PHYH |
| chr10 | 13325639 | 13325889 | 7 | 2 | PHYH |
| chr10 | 13330309 | 13330591 | 13 | 3 | PHYH |
| chr10 | 13333410 | 13333962 | 18 | 10 | PHYH |
| chr10 | 13336377 | 13337037 | 27 | 8 | PHYH |
| chr10 | 13337177 | 13337656 | 15 | 4 | PHYH |
| chr10 | 13340136 | 13340295 | 8 | 4 | PHYH |
| chr10 | 13341369 | 13341813 | 21 | 8 | PHYH |
| chr10 | 13341917 | 13342180 | 6 | 3 | PHYH |
| chr10 | 13343935 | 13344462 | 13 | 5 | PHYH |
| chr10 | 90519901 | 90520057 | 5 | 2 | LIPN |
| chr10 | 90521104 | 90521320 | 1 | 0 | LIPN |
| chr10 | 90521894 | 90522112 | 5 | 1 | LIPN |
| chr10 | 90524116 | 90524415 | 8 | 3 | LIPN |
| chr10 | 90525975 | 90526185 | 3 | 1 | LIPN |
| chr10 | 90528498 | 90528735 | 11 | 5 | LIPN |
| chr10 | 90530551 | 90530798 | 9 | 4 | LIPN |
| chr10 | 90534179 | 90534351 | 2 | 2 | LIPN |
| chr10 | 90534798 | 90534970 | 7 | 4 | LIPN |
| chr10 | 90537715 | 90538049 | 14 | 7 | LIPN |
| chr11 | 130029406 | 130030139 | 18 | 7 | ST14 |
| chr11 | 130057958 | 130058218 | 11 | 4 | ST14 |
| chr11 | 130058374 | 130058602 | 9 | 8 | ST14 |
| chr11 | 130058713 | 130058884 | 7 | 2 | ST14 |
| chr11 | 130059169 | 130059841 | 27 | 12 | ST14 |
| chr11 | 130059885 | 130060639 | 32 | 11 | ST14 |
| chr11 | 130063993 | 130064233 | 10 | 3 | ST14 |
| chr11 | 130064484 | 130064682 | 10 | 4 | ST14 |
| chr11 | 130066183 | 130066393 | 11 | 3 | ST14 |
| chr11 | 130066414 | 130066739 | 12 | 3 | ST14 |
| chr11 | 130067685 | 130067890 | 12 | 5 | ST14 |
| chr11 | 130068152 | 130068566 | 24 | 9 | ST14 |
| chr11 | 130068791 | 130069014 | 11 | 3 | ST14 |
| chr11 | 130069795 | 130070082 | 8 | 2 | ST14 |
| chr11 | 130078254 | 130078629 | 12 | 3 | ST14 |
| chr11 | 130079286 | 130080406 | 35 | 13 | ST14 |
| chr12 | 53038291 | 53039403 | 47 | 18 | KRT2 |
| chr12 | 53040473 | 53040794 | 13 | 3 | KRT2 |
| chr12 | 53041463 | 53041689 | 7 | 2 | KRT2 |
| chr12 | 53041906 | 53042171 | 12 | 4 | KRT2 |
| chr12 | 53042740 | 53042936 | 11 | 4 | KRT2 |
| chr12 | 53043647 | 53043808 | 6 | 2 | KRT2 |
| chr12 | 53044072 | 53044506 | 12 | 1 | KRT2 |
| chr12 | 53045291 | 53046009 | 32 | 13 | KRT2 |
| chr12 | 53068469 | 53069578 | 48 | 12 | KRT1 |
| chr12 | 53070008 | 53070329 | 14 | 4 | KRT1 |
| chr12 | 53070792 | 53071018 | 5 | 2 | KRT1 |

| | | | | | |
|---|---|---|---|---|---|
| chr12 | 53071049 | 53071351 | 9 | 2 | KRT1 |
| chr12 | 53071383 | 53071579 | 4 | 1 | KRT1 |
| chr12 | 53071896 | 53072057 | 5 | 0 | KRT1 |
| chr12 | 53072275 | 53072590 | 6 | 2 | KRT1 |
| chr12 | 53073491 | 53074241 | 32 | 13 | KRT1 |
| chr13 | 20761553 | 20763969 | 99 | 32 | GJB2 |
| chr13 | 20766871 | 20767164 | 6 | 5 | GJB2 |
| chr13 | 20796050 | 20797684 | 56 | 17 | GJB6 |
| chr13 | 20803668 | 20803938 | 8 | 3 | GJB6 |
| chr13 | 20804786 | 20805422 | 21 | 9 | GJB6 |
| chr13 | 20805470 | 20805694 | 4 | 2 | GJB6 |
| chr13 | 20806282 | 20806584 | 6 | 4 | GJB6 |
| chr13 | 29233090 | 29233374 | 17 | 5 | POMP |
| chr13 | 29233908 | 29234106 | 6 | 3 | POMP |
| chr13 | 29236496 | 29236694 | 5 | 1 | POMP |
| chr13 | 29238595 | 29238756 | 1 | 0 | POMP |
| chr13 | 29242559 | 29242761 | 6 | 1 | POMP |
| chr13 | 29246425 | 29246619 | 5 | 0 | POMP |
| chr13 | 29252121 | 29253143 | 26 | 4 | POMP |
| chr14 | 24718269 | 24718797 | 13 | 7 | TGM1 |
| chr14 | 24723278 | 24723544 | 11 | 3 | TGM1 |
| chr14 | 24723819 | 24724080 | 9 | 6 | TGM1 |
| chr14 | 24724127 | 24724509 | 13 | 3 | TGM1 |
| chr14 | 24724519 | 24724773 | 12 | 5 | TGM1 |
| chr14 | 24725144 | 24725333 | 4 | 0 | TGM1 |
| chr14 | 24727440 | 24727644 | 4 | 1 | TGM1 |
| chr14 | 24727690 | 24727929 | 9 | 4 | TGM1 |
| chr14 | 24728230 | 24728505 | 12 | 5 | TGM1 |
| chr14 | 24728859 | 24729067 | 10 | 3 | TGM1 |
| chr14 | 24729095 | 24729414 | 13 | 7 | TGM1 |
| chr14 | 24729605 | 24729954 | 18 | 1 | TGM1 |
| chr14 | 24730850 | 24731139 | 21 | 10 | TGM1 |
| chr14 | 24731189 | 24731610 | 28 | 12 | TGM1 |
| chr14 | 24732244 | 24732796 | 24 | 10 | TGM1 |
| chr14 | 24733089 | 24733688 | 15 | 6 | TGM1 |
| chr15 | 91541595 | 91542334 | 27 | 8 | VPS33B |
| chr15 | 91542510 | 91543073 | 20 | 4 | VPS33B |
| chr15 | 91543079 | 91543255 | 3 | 2 | VPS33B |
| chr15 | 91543669 | 91544285 | 16 | 8 | VPS33B |
| chr15 | 91544568 | 91544742 | 1 | 1 | VPS33B |
| chr15 | 91545229 | 91545462 | 8 | 2 | VPS33B |
| chr15 | 91546264 | 91546411 | 3 | 1 | VPS33B |
| chr15 | 91548056 | 91548211 | 5 | 1 | VPS33B |
| chr15 | 91548234 | 91548399 | 6 | 0 | VPS33B |
| chr15 | 91548559 | 91548734 | 6 | 4 | VPS33B |
| chr15 | 91548873 | 91549064 | 7 | 2 | VPS33B |
| chr15 | 91549152 | 91549339 | 7 | 2 | VPS33B |
| chr15 | 91549551 | 91550326 | 28 | 10 | VPS33B |
| chr15 | 91550648 | 91550853 | 6 | 1 | VPS33B |
| chr15 | 91551049 | 91551244 | 6 | 0 | VPS33B |
| chr15 | 91552980 | 91553126 | 3 | 0 | VPS33B |
| chr15 | 91556951 | 91557151 | 9 | 3 | VPS33B |
| chr15 | 91557563 | 91557713 | 5 | 3 | VPS33B |

| | | | | | |
|---|---|---|---|---|---|
| chr15 | 91560142 | 91560344 | 7 | 3 | VPS33B |
| chr15 | 91560984 | 91561165 | 5 | 0 | VPS33B |
| chr15 | 91561197 | 91561316 | 2 | 1 | VPS33B |
| chr15 | 91565333 | 91565883 | 20 | 7 | VPS33B |
| chr15 | 100940549 | 100943120 | 93 | 29 | CERS3 |
| chr15 | 100996047 | 100996301 | 11 | 7 | CERS3 |
| chr15 | 101009532 | 101009739 | 7 | 2 | CERS3 |
| chr15 | 101013078 | 101013307 | 11 | 5 | CERS3 |
| chr15 | 101015991 | 101016433 | 12 | 3 | CERS3 |
| chr15 | 101019582 | 101019751 | 4 | 1 | CERS3 |
| chr15 | 101020143 | 101020301 | 0 | 0 | CERS3 |
| chr15 | 101024704 | 101024923 | 10 | 2 | CERS3 |
| chr15 | 101030926 | 101031186 | 8 | 5 | CERS3 |
| chr15 | 101040402 | 101040759 | 10 | 3 | CERS3 |
| chr15 | 101041831 | 101042105 | 16 | 4 | CERS3 |
| chr15 | 101043864 | 101044103 | 10 | 4 | CERS3 |
| chr15 | 101061821 | 101062011 | 2 | 1 | CERS3 |
| chr15 | 101068967 | 101069520 | 15 | 10 | CERS3 |
| chr15 | 101084278 | 101084668 | 15 | 8 | CERS3 |
| chr15 | 101084805 | 101084975 | 7 | 2 | CERS3 |
| chr15 | 101085057 | 101085250 | 11 | 3 | CERS3 |
| chr17 | 7975903 | 7976318 | 8 | 5 | ALOX12B |
| chr17 | 7976415 | 7976686 | 5 | 1 | ALOX12B |
| chr17 | 7976924 | 7977125 | 6 | 3 | ALOX12B |
| chr17 | 7978862 | 7979084 | 7 | 5 | ALOX12B |
| chr17 | 7979442 | 7979712 | 11 | 3 | ALOX12B |
| chr17 | 7979924 | 7980111 | 1 | 0 | ALOX12B |
| chr17 | 7980257 | 7980561 | 13 | 6 | ALOX12B |
| chr17 | 7982663 | 7982907 | 6 | 3 | ALOX12B |
| chr17 | 7983036 | 7983463 | 10 | 5 | ALOX12B |
| chr17 | 7983502 | 7983706 | 9 | 1 | ALOX12B |
| chr17 | 7983925 | 7984148 | 5 | 2 | ALOX12B |
| chr17 | 7984151 | 7984344 | 12 | 3 | ALOX12B |
| chr17 | 7984373 | 7984555 | 5 | 2 | ALOX12B |
| chr17 | 7989283 | 7989588 | 13 | 6 | ALOX12B |
| chr17 | 7990563 | 7991072 | 20 | 8 | ALOX12B |
| chr17 | 7999167 | 8000174 | 40 | 14 | ALOXE3 |
| chr17 | 8006590 | 8006883 | 10 | 2 | ALOXE3 |
| chr17 | 8007382 | 8007583 | 4 | 2 | ALOXE3 |
| chr17 | 8011735 | 8011957 | 7 | 0 | ALOXE3 |
| chr17 | 8012441 | 8012711 | 9 | 4 | ALOXE3 |
| chr17 | 8013183 | 8013896 | 23 | 4 | ALOXE3 |
| chr17 | 8014626 | 8014899 | 8 | 1 | ALOXE3 |
| chr17 | 8015360 | 8015564 | 4 | 1 | ALOXE3 |
| chr17 | 8017751 | 8017977 | 15 | 3 | ALOXE3 |
| chr17 | 8018205 | 8018425 | 7 | 3 | ALOXE3 |
| chr17 | 8018874 | 8019056 | 3 | 3 | ALOXE3 |
| chr17 | 8020043 | 8020348 | 13 | 3 | ALOXE3 |
| chr17 | 8021111 | 8021910 | 17 | 9 | ALOXE3 |
| chr17 | 8021963 | 8022415 | 19 | 7 | ALOXE3 |
| chr17 | 19551398 | 19551628 | 5 | 3 | ALDH3A2 |
| chr17 | 19551788 | 19552487 | 28 | 10 | ALDH3A2 |
| chr17 | 19554809 | 19555141 | 8 | 0 | ALDH3A2 |

| | | | | | |
|---|---|---|---|---|---|
| chr17 | 19555809 | 19556060 | 8 | 1 | ALDH3A2 |
| chr17 | 19558543 | 19558750 | 3 | 1 | ALDH3A2 |
| chr17 | 19559407 | 19559937 | 17 | 4 | ALDH3A2 |
| chr17 | 19560983 | 19561225 | 4 | 1 | ALDH3A2 |
| chr17 | 19562835 | 19563062 | 8 | 6 | ALDH3A2 |
| chr17 | 19564389 | 19564805 | 10 | 2 | ALDH3A2 |
| chr17 | 19566595 | 19566865 | 5 | 2 | ALDH3A2 |
| chr17 | 19568210 | 19568410 | 8 | 2 | ALDH3A2 |
| chr17 | 19574983 | 19575319 | 6 | 1 | ALDH3A2 |
| chr17 | 19575614 | 19575794 | 3 | 3 | ALDH3A2 |
| chr17 | 19576413 | 19576638 | 6 | 0 | ALDH3A2 |
| chr17 | 19577346 | 19580961 | 81 | 20 | ALDH3A2 |
| chr17 | 38974318 | 38974784 | 14 | 6 | KRT10 |
| chr17 | 38974988 | 38975463 | 15 | 6 | KRT10 |
| chr17 | 38975718 | 38976093 | 6 | 2 | KRT10 |
| chr17 | 38976250 | 38976969 | 15 | 3 | KRT10 |
| chr17 | 38977236 | 38977419 | 4 | 0 | KRT10 |
| chr17 | 38978160 | 38978913 | 28 | 9 | KRT10 |
| chr19 | 15619285 | 15619444 | 4 | 3 | CYP4F22 |
| chr19 | 15634453 | 15634660 | 4 | 2 | CYP4F22 |
| chr19 | 15636093 | 15636419 | 19 | 9 | CYP4F22 |
| chr19 | 15640469 | 15640714 | 9 | 2 | CYP4F22 |
| chr19 | 15648121 | 15648275 | 7 | 3 | CYP4F22 |
| chr19 | 15648295 | 15648523 | 8 | 4 | CYP4F22 |
| chr19 | 15648632 | 15648854 | 11 | 4 | CYP4F22 |
| chr19 | 15651210 | 15651578 | 24 | 8 | CYP4F22 |
| chr19 | 15654731 | 15654898 | 5 | 1 | CYP4F22 |
| chr19 | 15654910 | 15655140 | 2 | 0 | CYP4F22 |
| chr19 | 15658868 | 15659102 | 10 | 3 | CYP4F22 |
| chr19 | 15659898 | 15660063 | 5 | 2 | CYP4F22 |
| chr19 | 15661434 | 15661617 | 2 | 2 | CYP4F22 |
| chr19 | 15662054 | 15663178 | 33 | 15 | CYP4F22 |
| chr19 | 45854195 | 45855029 | 64 | 21 | ERCC2 |
| chr19 | 45855416 | 45855660 | 19 | 5 | ERCC2 |
| chr19 | 45855713 | 45856124 | 24 | 4 | ERCC2 |
| chr19 | 45856290 | 45856642 | 20 | 6 | ERCC2 |
| chr19 | 45857937 | 45858159 | 10 | 2 | ERCC2 |
| chr19 | 45858872 | 45859036 | 5 | 1 | ERCC2 |
| chr19 | 45860477 | 45860679 | 8 | 1 | ERCC2 |
| chr19 | 45860681 | 45861007 | 11 | 2 | ERCC2 |
| chr19 | 45861972 | 45862220 | 16 | 5 | ERCC2 |
| chr19 | 45864731 | 45864950 | 9 | 3 | ERCC2 |
| chr19 | 45866950 | 45867427 | 19 | 4 | ERCC2 |
| chr19 | 45867442 | 45867855 | 19 | 7 | ERCC2 |
| chr19 | 45868045 | 45868466 | 14 | 5 | ERCC2 |
| chr19 | 45871837 | 45872051 | 9 | 1 | ERCC2 |
| chr19 | 45872137 | 45872455 | 8 | 2 | ERCC2 |
| chr19 | 45873340 | 45873926 | 20 | 5 | ERCC2 |
| chr22 | 21213220 | 21213685 | 26 | 7 | SNAP29 |
| chr22 | 21213720 | 21213978 | 6 | 2 | SNAP29 |
| chr22 | 21224574 | 21225379 | 21 | 7 | SNAP29 |
| chr22 | 21235286 | 21235472 | 5 | 2 | SNAP29 |
| chr22 | 21237708 | 21237907 | 3 | 0 | SNAP29 |

| | | | | | |
|---|---|---|---|---|---|
| chr22 | 21241916 | 21245552 | 132 | 38 | SNAP29 |
